# An overview of systematic reviews of economic evaluations of pharmacy-based public health interventions: addressing methodological challenges

**DOI:** 10.1186/s13643-019-1177-3

**Published:** 2019-11-11

**Authors:** Suzete Costa, Maria Cary, Dennis K. Helling, João Pereira, Céu Mateus

**Affiliations:** 1 Escola Nacional de Saúde Pública (ENSP), Universidade NOVA de Lisboa, Avenida Padre Cruz, 1600-560 Lisbon, Portugal; 2 USFarmácia® Collaborative Care Project, Associação Nacional das Farmácias, R. Marechal Saldanha, 1, 1249-069 Lisbon, Portugal; 3 Institute for Evidence-Based Health (ISBE), Faculdade de Medicina da Universidade de Lisboa, Av. Prof. Egas Moniz, 1649-028 Lisbon, Portugal; 4 Centre for Health Evaluation & Research (CEFAR), Associação Nacional das Farmácias, R. Marechal Saldanha, 1, 1249-069 Lisbon, Portugal; 5 University of Colorado Skaggs School of Pharmacy and Pharmaceutical Sciences, 8189 East 5th Avenue, Denver, CO 80230 USA; 6 Centro de Investigação em Saúde Pública (CISP), Avenida Padre Cruz, 1600-560 Lisbon, Portugal; 7 Division of Health Research, Health Economics at Lancaster, Lancaster University, Lancaster, LA1 4YG UK

**Keywords:** Overview, Umbrella review, Systematic review, Economic evaluation, Cost-effectiveness, Community pharmacy, Pharmacy interventions, Pharmacy services, Public health interventions, Methods

## Abstract

**Background:**

Pharmacy interventions are a subset of public health interventions and its research is usually performed within the scope of a trial. The economic evaluation of pharmacy interventions requires certain considerations which have some similarities to those of public health interventions and to economic evaluations alongside trials. The objective of this research is to perform an overview of systematic reviews of economic evaluations of pharmacy services and triangulate results with recommendations for economic evaluations of both public health interventions and alongside trials.

**Methods:**

(1) Exploratory review of recommendations on the economic evaluation of public health interventions, (2) exploratory review of recommendations for conducting economic evaluations alongside trials, (3) overview of systematic reviews of economic evaluations of pharmacy interventions (protocol registered with PROSPERO 2016 outlining information sources, inclusion criteria, appraisal of reviews and synthesis methods).

**Results:**

Fourteen systematic reviews containing 75 index publications were included. Reviews reported favorable economic findings for 71% of studies with full economic evaluations. The types of economic analysis are diverse. Two critical quality domains are absent from most reviews. Key findings include the following: certain types of risk of bias, wider scope of study designs, and most economic quality criteria met but some issues unresolved or unclear. Triangulation revealed additional gaps. Limitations include choice of critical quality domains and potential biases in the overview process.

**Conclusions:**

Economic evaluations of pharmacy-based public health interventions seem to follow most economic quality criteria, but there are still some issues in certain key areas to improve. These findings may assist in improving the design of pilot trials of economic evaluations in pharmacy, leading to robust evidence for payers. Based on the findings, we propose a methodological approach for the economic evaluation of pharmacy-based public health interventions.

**Systematic review registration:**

PROSPERO CRD42016032768

## Background

Pharmacy-based public health interventions can be defined as complex health interventions, provided by pharmacists to patients in the community pharmacy setting, which are beyond, but do not necessarily exclude, the medication supply role.

These interventions include health promotion and support on self-monitoring, disease prevention, screening, and disease and medication management and cover a wide spectrum of areas, including the main public health areas of interest as defined by the National Institute for Health and Care Excellence (NICE): cardiovascular disease; chronic illness; diabetes; drugs; mental health; obesity; physical activity; screening; sexual health; smoking and tobacco; and vaccine preventable diseases [1], to name only the more relevant.

Complex health interventions require several interacting components, including behavioral changes from providers and patients, factors influencing multiple levels, and some degree of flexibility of interventions [2]. All these features have also been identified in pharmacy interventions [3, 4].

The strategies used to operate pharmacy-based complex health interventions at multiple levels seem to be consistent with the diffusion of innovation theory [5]. At the patient level, behavioral changes seem to be consistent with the theory of planned behavior [6] for screening; the information-motivational-behavioral skills model for medication adherence [7]; and social cognitive theory, the transtheoretical model, and the theory of planned behavior for disease management [8]. Behavior changes in pharmacists and in patients then lead to improved health outcomes [4]. The conceptual logic framework, used by Blalock et al., and a logic model, as per the Medical Research Council (MRC), are interesting avenues to explore [4, 9]. These interventions fall under the umbrella of prevention interventions, as opposed to treatment interventions. However, according to the Organization for Economic Co-operation and Development (OECD), in 2010, only 3% of health budgets in the European Union (EU) were spent on prevention, yet a good balance in financing of treatment and prevention was found to be cost-effective to improve health outcomes [10].

Financing prevention interventions requires economic evaluations which should be a routine and consistent part of all public health interventions [11], including pharmacy-based public health interventions.

In fact, the original definition of pharmacoeconomics clearly identified the description and analysis of the costs and consequences of pharmacy services as a domain of pharmacoeconomics [12]. However, the economic evaluation of pharmacy services requires certain unique considerations [13]. Many of these challenges seem to involve comparators, selection bias, social interaction threats, outcome measures, study design, effect of interventions, type of economic evaluation, and study site [13, 14]. Some of these issues do not seem to be exclusive to pharmacy; they also exist in public health interventions [15]. For instance, the Centers for Disease Control and Prevention (CDC) and NICE have acknowledged these major specificities [1, 16]. The MRC also underlines that the economic evaluation is one of the key components of the development and evaluation of complex health interventions [2].

In addition, there are also methodological recommendations for conducting economic evaluations alongside clinical or pragmatic trials, which may be useful to explore [17, 18].

The first economic evaluations of clinical pharmacy services, a subset of public health interventions provided by pharmacists to optimize medication therapy and promote health, wellness, and disease prevention, were published in the late 1970s. As the profession progressed beyond dispensing, an increasing number of evaluations were published [13].

A landmark economic study demonstrated the important healthcare savings of medication management services provided by pharmacists [19]. This landmark study was still restricted to a clinical pharmacy service. Later on, evidence of health outcome improvements in broader public health interventions provided by community pharmacists in appropriate collaborative environments with physicians was established [4, 20–22]. In the UK, the effectiveness and cost-effectiveness of the contribution of community pharmacy to improving public health has been established for certain pharmacy interventions beyond clinical pharmacy services [23].

Hence, the economic evaluation of such services in collaborative care environments may contribute to informing decisions made regarding the successful expansion, continuation, or justification of such services [13].

The overall aim of this research is to perform an overview of systematic reviews of economic evaluations of pharmacy services to better understand their successes and downsides and to assist in improving the methods for future research, following the early work of Schumock [13, 14] and recent important contributions by Elliott et al. [24], but focusing on the community pharmacy setting.

Since the term “pharmacy services” is more frequently used by researchers in pharmacy practice and the term “public health interventions” is, however, used more frequently in the context of health technology assessment and economic evaluation, we will use the terms pharmacy-based public health interventions and pharmacy services interchangeably.

There are already a few systematic reviews on the economic evaluation of pharmacy services, which support the rationale to conduct this overview of reviews. We found two overviews of reviews on pharmacy services [21, 22]. However, they address the question of effectiveness and are not focused on methods.

This overview is, to the best of our knowledge, the first centered on methods of economic evaluations exclusively in the pharmacy setting and triangulating results with recommendations for economic evaluations of public health interventions and for economic evaluations alongside trials.

## Objectives

The objectives of this paper are as follows: to review the methods and issues in systematic reviews of economic evaluations of pharmacy services compared with usual care or other alternatives in multiple population groups; to compare these with recommendations on the economic evaluation of public health interventions and conducted alongside clinical and pragmatic trials; to propose a system for the measurement and valuation of costs and health effects feasible for the economic evaluation studies of pharmacy services; and to contribute to the methods in the economic evaluation of pharmacy services and of public health interventions.

## Methods

### Exploratory review of recommendations on the economic evaluation of public health interventions

We selected four policy documents that addressed the specifics and issued recommendations on the economic evaluation of public health interventions: Kelly et al. [11], Honeycutt et al. [16], MRC [2], and NICE [1]. Six academic papers were also selected: Cookson et al. [25], Weatherly et al. [15], Lorgelly et al. [26], Marsh et al. [27], Edwards et al. [28], and Alayli-Goebbels et al. [29].

This was a selected search on existing recommendations on the economic evaluation of public health interventions with the purpose of obtaining key insights for triangulation. This selected search was performed on MEDLINE® (via PubMed) in titles/abstracts till July 2017. A first search used the following terms in titles/abstracts: “review” and “economic evaluation” and “public health interventions” and “methods” which returned 15 titles. After reviewing title and abstract, we included five relevant articles [15, 25–28]. A second similar search was performed replacing “public health interventions” for “behavior” and “interventions” which returned 22 titles of which, after reviewing title and abstract, we selected one [29]. We then performed snowballing from reference lists of more recent included articles [28, 29] and further selected four policy papers [1, 2, 11, 16].

### Exploratory review of recommendations for conducting economic evaluations alongside clinical or pragmatic trials

We selected three academic publications that issued specific recommendations for conducting economic evaluations alongside clinical or pragmatic trials: O’Sullivan et al. [17], Petrou and Gray [18], and Ramsey et al. [30].

This was a selected search on existing recommendations on the economic evaluation alongside clinical or pragmatic trials with the purpose of obtaining key insights for triangulation. This selected search was first performed on MEDLINE® (via PubMed) till July 2017 using the term “economic evaluation” in title/abstract and the following terms in title: “alongside” and (“clinical trials” or “randomized controlled trials”). This first search returned 14 titles of which we just selected the two most recent relevant titles available for free [17, 18]. We then performed a search on ISPOR website under Good Practices for Outcomes Research on July 2017, and we selected the most recent report on economic evaluation alongside clinical trials [30].

### Overview of systematic reviews of economic evaluations of pharmacy interventions

This overview considered recommendations by Cochrane, by Smith et al., and by the Joanna Briggs Institute (JBI) [31–33] for overviews and by the Centre for Reviews and Dissemination (CRD) of the University of York and Cochrane for systematic reviews for public health interventions and for economic evaluations [34–36].

We have followed PRISMA Checklist in reporting this overview. See supplementary completed PRISMA Checklist, Additional file 1.

#### Protocol registration and eligibility criteria

The protocol of this overview was registered with the International Prospective Register of Systematic Reviews (PROSPERO) on 7 January 2016. Reference number PROSPERO 2016: CRD42016032768 (http://www.crd.york.ac.uk/PROSPERO/display_record.asp?ID=CRD42016032768). It outlines the methods for eligibility criteria, information sources, search strategy, study selection, data extraction, quality assessment, and strategy for data synthesis. Conference abstracts (no full papers available) and study protocols were excluded.

Reviews were included if they met the following inclusion criteria: systematic review as per stated by authors or implied by methods; describing or containing individual economic evaluation studies (economic evaluation and/or equivalent terms “economic” OR “economic evaluation” OR “cost-effectiveness” OR “cost-utility” OR “cost-benefit” explicitly stated in search terms of systematic review); describing pharmacist-provided patient care interventions as per protocol definition published in PROSPERO (complex public health interventions, in health promotion, disease prevention, and disease/medication management, provided by pharmacists to patients in the community pharmacy setting, with the aim of preventing disease, promoting health, and prolonging life, which are beyond, but not necessarily excluding, the medication supply role); and describing or containing at least one economic study in community pharmacy setting.

No restriction on the types of populations, comparators, nor outcomes was considered, as stated in protocol, as this was an inclusive review to critique methods and aid future research design.

#### Information sources

A comprehensive search was performed in the following databases through July 2017: MEDLINE®In-Process & Other Non-Indexed Citations, MEDLINE® (from 1946) and EMBASE (from 1980), via the OVID SP interface; Cochrane Database of Systematic Reviews (CDSR); Database of Abstracts of Reviews of Effects (DARE), NHS Economic Evaluation Database (NHS EED) and HTA, via the CRD database; Tufts CEA Registry; and Web of Science to identify existing systematic reviews of economic evaluation studies of pharmacy services.

In addition, a further search was performed in Google Scholar and snowballing from reference lists of retrieved reviews. An additional search for gray literature was performed in the ISPOR Database and OpenGrey.

Finally, a search for ongoing systematic reviews was performed in PROSPERO.

#### Search

Searches used the following terms: “systematic review” and “economic evaluation” (or “cost-effectiveness” or “cost-utility” or “cost-benefit”) and “pharmacy” (or “pharmacist”) and “intervention” (or “service” or “program” or “management”) with slight changes, according to the conventions of each database, in combination with database-specific filters for systematic reviews and economic evaluations.

See supplementary detailed search strategies, Additional file 2.

#### Selection of reviews

Citations that resulted from searches were downloaded, and duplicates were removed. Two researchers (SC and MC) reviewed all potentially relevant titles against the inclusion criteria and reviewed the abstracts associated with retrieved titles. Finally, full-text articles of retrieved abstracts were reviewed for eligibility by two review team members (SC and CM). Disagreements were resolved through discussion with additional review team members (CM and DKH). We recorded the reasons for exclusion at screening.

See supplementary list of excluded reviews, Additional file 3.

We have followed the PRISMA flowchart in reporting the study selection [37].

#### Data collection process

A template adapted from the JBI Data Extraction Form for Reviews of Systematic Reviews [33] was developed and prepiloted to assist with data extraction.

Extracted data (SC) of eight randomly selected reviews were audited by two other review team members (CM and DKH). Existing discrepancies were resolved through discussion. None of the review team members was blind to journal titles or to study authors or institutions.

Since primary studies are often included in more than one review, the degree of overlap was determined using the corrected covered area (CCA) method of Pieper et al. A CCA value lower than 5% is a slight overlap [38].

#### Data items

The following items were extracted: title; first author/year of publication; journal; objectives; no. and sources searched; date range of reviews; no. of included economic evaluation community pharmacy (CP) studies/total no. of studies; countries of origin of CP studies; populations in CP studies; interventions in CP studies; comparators in CP studies; outcomes in CP studies; study designs in CP studies; types of economic evaluation in CP studies/total; cost and resource use categories in CP studies; cost year/discount rates in CP studies; data sources in CP studies; perspectives in CP studies; key findings in CP studies; uncertainty in included studies; assessment of quality of evidence for deriving effectiveness; assessment of risk of bias; assessment of economic quality; method of data synthesis; key findings of SR; significance/direction; assessment of heterogeneity; process indicators; equity considerations; sustainability of interventions; context; source of funding; conflict of interest; methodological challenges identified by authors; comments.

The list of references in the primary studies in the community pharmacy setting included in the systematic review was also recorded in this extraction form. Primary studies were not consulted. We extracted data of primary community pharmacy studies provided data were reported in the systematic review in accordance with guidelines on overviews.

#### Assessment of methodological quality of included reviews

A threefold critical appraisal of the methodology was performed as per protocol and further detailed.

##### Quality of included reviews

We used the 16-item Assessment of Multiple Systematic Reviews (AMSTAR 2) questionnaire to measure the general methodological quality of each included systematic review [39], assisted by a general critique on four critical domains considered: protocol registered before commencement of the review, adequacy of the literature search, justification for excluding individual studies, and consideration of risk of bias when interpreting the results of the review.

##### Quality of evidence of CP primary studies reported in reviews

We assessed the following: the overall quality of evidence for deriving effectiveness reported in systematic reviews assisted by the Cochrane guidelines “What study designs should be included in an EPOC review” [40] and “Suggested Risk of Bias Criteria for Effective Practice and Organization of Care (EPOC) Reviews” [41]; the quality of economic evaluations of the studies reported in systematic reviews assisted by the Consolidated Health Economic Evaluation Reporting Standards (CHEERS) checklist [42]; the heterogeneity of populations, interventions, and outcomes, as described by Mossialos et al. [21] and recommended by CRD for systematic reviews of public health interventions [34]; and the presence of wider research issues [9, 34, 36].

Quality assessment of primary studies relies on reported information in included reviews.

##### Applicability and transferability

All systematic reviews were then collectively assessed in terms of applicability and transferability of interventions, as described by Mossialos et al. [21], Wang et al. [43], and CRD and Cochrane [34–36].

Quality assessment was performed by the lead reviewer (SC). Quality of all included reviews, applicability, and transferability were reviewed by another review team member (MC). Quality of evidence in eight randomly selected included reviews was reviewed by two other review team members (CM and DKH). Disagreements were resolved through discussion.

#### Synthesis of results

Information on study characteristics from systematic reviews was drawn from the data extraction form and reported in evidence summary tables. A narrative synthesis was performed, but no additional statistical analysis was performed. Whenever results described multiple settings, we limited ourselves to the subset in the pharmacy setting.

Based on the findings, we proposed a methodological approach for the measurement and valuation of costs and health effects and for the types of analysis for economic evaluations of pharmacy interventions.

## Results

### Exploratory review of recommendations on the economic evaluation of public health interventions

First, a systematic review or, at least, an in-depth review of the best available evidence of the effectiveness and cost-effectiveness should be performed prior to the economic evaluation [1, 2]. Second, economic appraisal should be linked to the appraisal of effectiveness [1, 2, 11, 12]. Third, although individual or cluster randomized controlled trials (RCTs) are the preferred study design, they may not always be feasible in the economic evaluation of public health interventions, and randomization may be difficult, in which case quasi-experimental designs could be an option [2, 11] since few RCTs exist or are not feasible or appropriate [15, 26–29]. Weatherly et al. also suggests the use of econometric techniques [15]. Marsh et al. proposes alternative study designs [27]. Fourth, a societal perspective should be adopted [2, 16]. NICE mentions the public sector’s perspective but acknowledges the societal perspective, where appropriate [1]. Fifth, costs and outcomes should be collected during trial or through decision analytic models but may need further modeling or estimation procedures based on links between measurable outcomes and long-term outcomes [1, 2, 11, 16] reinforced by academic papers [15, 26, 29]. Sixth, public health interventions tend to generate broad outcomes, which may not be captured by quality-adjusted life years (QALYs). Hence, the types of economic evaluations preferred for public health interventions are cost-benefit analysis (CBA), to capture broader health and nonhealth benefits [1, 11, 16], and cost-consequence analysis (CCA), due to the frequent nature of multiple outcomes in public health interventions [1, 11]. Cost-utility analysis (CUA) is also required by NICE whenever health is the sole or predominant benefit [1]. Both CUA and cost-effectiveness analysis (CMA) may be used as well [11, 16]. Seventh, academic papers also report that broad costs and benefits of public health interventions may fall to nonhealth sectors and need to be captured in economic evaluations [15, 28, 29]. Weatherly et al. mentions the intersectoral impacts in CCA and the general equilibrium approach [15]. In addition to CCA, Weatherly et al. also suggests CBA, CUA, and multiple-criteria decision analysis (MCDA) [15]. Lorgelly et al. suggests a capability to encompass health and nonhealth dimensions [26]. Marsh et al. also suggests CBA, capabilities, subjective well-being, MCDA, and better modeling [27]. Eighth, recommended discounting rates for both costs and benefits in economic evaluations of public health interventions are 1.5% for NICE [1] and 3% for CDC [16]. Ninth, equity considerations need to be included [1, 2, 11]. Academic papers also report equity considerations [15, 25, 26, 28, 29]. Cookson et al. suggests possible approaches [25]. Some of these approaches were also identified by Lorgelly et al. and Edwards et al. [26, 28]. Tenth, the economic evaluation of public health interventions should include a wider spectrum of research methods, including qualitative and quantitative research, to understand the contextual and process indicators affecting behavior change [1, 2, 11, 16].

### Exploratory review of recommendations for conducting economic evaluations alongside clinical or pragmatic trials

First, most authors consider that such economic evaluations should be based on well-designed pragmatic trials with fewer strict protocols [17, 18]. Second, selection of subjects and sites should also seek for proximity to real-world populations [17, 18]. Third, sample size should be based on important clinical outcomes believed to be correlated with economic outcomes [17, 18]. Fourth, an appropriate length of follow-up is required. Estimates beyond trial time horizon are important and require good modeling [17, 18]. Fifth, current practice or standard of care should be the comparator, although there may be different standards of care in the comparator [17, 18]. Sixth, outcome measures, if composite, should be disaggregated, and direct measures are preferred. Health state utilities should be collected directly from study subjects at regular intervals by instruments or mapping techniques [18, 30]. Petrou mentions that QALYs may sometimes be too restrictive or insensitive [18]. Seventh, most relevant or economically important resource use and cost measures should be collected together with clinical data recorded in case report forms (CRFs) or patient medical records, patient diaries, and/or interviews; computerized record linkage may also be an option in the future [17, 18]. Eighth, valuation in costs needs to be consistent with resource use, perspective, and time horizon. It may include microcosting, unit costing, and gross costing [17, 18]. Ninth, papers outline several additional recommendations concerning methods: plan of statistical analysis and hypothesis prior to trial; plan for on-going data quality monitoring; incremental analysis with an intention-to-treat approach; common time horizon for costs and outcomes; within-trial assessment of costs and outcomes; arithmetic mean cost differences for cost comparisons and bootstrapping, OLS or GLM to compare difference between groups; multivariable methods for analysis of outcomes; uncertainty through confidence intervals, *p* values, and incremental cost-effectiveness ratios (ICERs) on various time horizons; common discount rate; accounting for missing/censored data through imputation of missing data using multiple imputation approaches; and one or more summary measures (ratio, difference, and/or probability measures) [18, 30]. Tenth, Ramsey and Petrou mention that reporting should include the following: description of trial; major findings; economic data collected; missing and censored data; methods to project costs and outcomes; statistical methods; resource use, costs and outcomes; and results within and beyond time horizon of trial [18, 30].

### Overview of systematic reviews of economic evaluations of pharmacy interventions

#### Study selection

Electronic searches until July 2017 identified 761 potential citations, of which 45 duplicates were found and removed, leaving 716 potential titles. The initial title screening excluded 594 (588 not matching inclusion criteria, 4 duplicates, and 2 protocols) titles, leaving a total of 122 potentially relevant titles. Abstract assessment resulted in the further exclusion of 100 studies and 22 potentially relevant abstracts were retrieved. The full-text assessment process resulted in 14 articles being retrieved.

OpenGrey and ISPOR Databases returned 1 and 77 potential titles, respectively, but all were excluded after abstract assessment. Hence, no references were obtained from gray literature.

Snowballing did not identify further reviews. A total of 14 reviews were included [24, 44–56].

PROSPERO registry returned 28 potential titles of ongoing reviews which were excluded after abstract assessment.

**Fig. 1:**
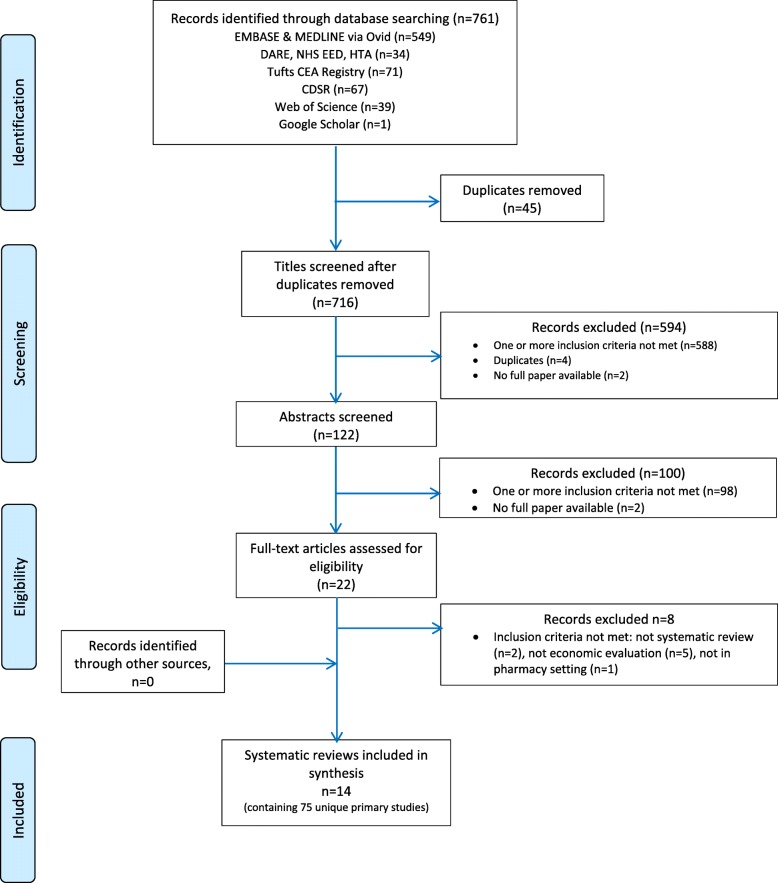
Study selection process

Figure 1 illustrates the study selection process.

#### Description of included reviews

Fourteen systematic reviews containing 118 included publications (including double counting) corresponding to 75 index publications on economic evaluation in community pharmacy were included.

A CCA value of 4.4% was obtained, indicating only a slight degree of overlap.

##### Characteristics of included reviews

Search dates of systematic reviews range from 1988 to 2015 (28 years).

Reviews were published in 11 different journals: 5 pharmacy journals (Pharmacotherapy, American Journal of Health-System Pharmacists, Journal of Managed Care Pharmacy, Journal of Clinical Pharmacy and Therapeutics, Annals of Pharmacotherapy); 2 health journals (BMJ Open, Health Policy); 2 pharmacoeconomic journals (Pharmacoeconomics, Expert Review of Pharmacoeconomics & Outcomes Research); and 2 disease-specific journals (Diabetic Medicine, Hellenic Journal of Cardiology).

Only two reviews exclusively focused on both economic evaluation studies and the community pharmacy setting [53, 55]. Economic evaluation studies in community pharmacy represented 87.5% of studies in Altowaijiri et al. [49], 71% in Gammie et al. [56], 45% in Elliot et al. [24], and 40% in Wang et al. [51].

Reviews contain studies from 12 different countries, of which 7 are from Europe. The origin of studies in reviews published until 2013 was mostly the USA and other countries outside of Europe. After 2014, we see an increase in the proportion of studies from Europe.

With a few exceptions [49, 51, 52, 54], most reviews tend to include studies addressing all very diverse populations, interventions, and outcomes. Populations do not seem to be well detailed.

Eight reviews allowed randomized trials, nonrandomized trials, and other controlled designs. Only 3 reviews reported RCT as inclusion criteria for included studies [49, 54, 55].

Table 1 summarizes the characteristics of included reviews.

**Table 1:** Characteristics of included reviews

First author (year SR)	No. databases	Search until	No. included studies (CP/total)	Countries of origin in CP studies	Populations in CP studies	Interventions in CP studies	Comparators in CP studies	Outcomes in CP studies	Study design in trial-based CP studies
Schumock GY (1996) [44]	2	1988–1995	2/104 (1.9%)	USA (2)	Patients requiring therapeutic monitoring	Therapeutic monitoring	None	Drug costs avoided and no. interventions; avoided medical care costs per intervention	–
Schumock GY (2003) [45]	2	1996–2000	6/59 (10.1%)	USA (5), Australia (1)	Patients individuals: requiring therapeutic monitoring; DS; with drug related problems; for flu immunization.	Therapeutic monitoring; DSM; patient education or cognitive service; flu immunization	Yes (4)	Changes in physician office visits, prescriptions and charges; medical and prescription utilizations and costs; costs per prescription; healthcare utilization cost savings	4 (controlled) trials
Perez A (2008) [46]	2	2001–2005	16/93 (17%)	USA (5), Australia (4), UK (3), Canada (3), Multicentric (1)	Total sample 13,304 patients in 15 studies (median 181): requiring therapeutic monitoring; DS; with dose related problems; for flu immunization; smokers.	MTM (1); therapeutic monitoring (4); DSM (9); dose optimization (1); flu immunization (1); smoking cessation (1). Median length of follow-up: 9 months.	Yes (8)	Changes in medical costs (most studies); adherence, knowledge and satisfaction; QALYs	4 randomized trials + 1 (controlled) + 3 nonrandomized
Chisholm Burns MA (2010) [47]	13	Jan/09	1/20 (126 total) (5%)	USA (1)	NR	Diabetes care	No	Changes in HbA1c and in cholesterol	–
Touchette DR (2014) [48]	4	2006–2010	8/25 (32%)	UK (5), USA (3)	Total sample 7134 patients in 7 studies (median 760): DS; requiring therapeutic monitoring > 75 years.	DSM (6); therapeutic monitoring (1). Median length of follow-up: 12 months.	Yes (6)	Changes in no. medicines, compliance; antiplatelet drug prescribing, CV disease visits; BP, LDL-C, HDL, TG, CV rate; % patients with asthma action plans, ED visits, hospital admissions; HbA1c, LDL-C, BP, influenza vaccination rate, eye and foot exam rate; MAI score, number of drugs	3 randomized + 1 multiple interrupted time series + 1 before/after
Altowaijri A (2013) [49]	13	Feb 2011	7/8 (87.5%)	UK (3), Canada (2), Australia (1), Thailand (1)	Total sample size NR. Patients: DS; smokers.	CV: DSM (4) and CV risk smoking cessation (3). Median length of follow-up reported in 3 studies: 9 months.	Yes (all)	Changes in CHD patients’ outcomes; CV risk; no. quitters; HbA1c, glucose; probability of events	Most seem to be trials (2 randomized + 1 nonrandomized, 1 controlled)
Elliott RA (2014) [24]	2	2003–2013	14/31 (45%)	UK (8), The Netherlands (3), USA (1), Australia (1), Canada (1)	Total sample > 7000 patients in 7 studies: DS; elderly/on high no. medicines; smokers.	DSM (6); MTM (8); smoking cessation (2); screening (2). Follow-up interventions in 5 studies: 6 months (2), 6–12 months (2), 1–2 years (1).	Yes (13, 7 well described)	Changes in adherence; prescribing errors/inappropriate prescribing; medication changes; infection, disease, quit rates); CV indicators; frequency of ED or hospital admissions; utility	4 randomized + 5 cluster randomized + 1 nonrandomized + 1 multiple interrupted time series
Brown TJ (2016) [50]	10	May 2014	4/19 (21%)	UK (3), Australia (1)	Total sample size 2791 smokers > 21 cigarettes/day in 4 studies. Mean age 24–44. Females 54–68.7%. SES variables collected in some studies.	Smoking cessation. Follow-up per patient 26 or 52 weeks. Some studies reported Stages of Change Model.	Yes, usual care (advice + NRT) and other settings	Quit rate (self-reported or CO measurement or Fagerström Test)	2 randomized, 1 nonrandomized
Wang Y (2016) [51]	6	2006–2014	10/25 (40%)	USA (7), Canada (1), Australia (1), Bulgaria (1)	Total sample 1238 patients in 10 studies (median 68): diabetic patients (5 studies in type 2 diabetics).	Diabetes: DSM (8); medication review (2). Median length of follow-up: 12 months.	Yes (all)	Changes in medical costs (healthcare use)	3 randomized trials + 2 nonrandomized trials + 3 nonconcurrent cohorts + 1 retrospective cohort
Peletidi A (2016) [52]	7	1990–2014	2/6 (33.3%)	UK (2)	Total sample 3764 in 2 studies: majority female smokers ≥ 21 cigarettes/day.	Smoking cessation. Length of follow-up: 4 weeks or 12 week; measurement at 4 and at 52 weeks after quitting.	Yes, self-quit rate	Changes in CO-validated quitters drop in CO levels	–
Perraudin C (2016) [53]	5	2004–2015	21/21 (100%)	UK (13), The Netherlands (3), Spain (2), Belgium (1), France (1), Denmark (1)	Total sample size NR. Patients: at risk of serious medication errors; elderly on multiple medicines; new to therapy; DS; with minor ailments; smokers.	MTM (5); DSM (3); adherence (5); smoking cessation (5); screening (2); minor ailment (1). Follow-up 6 or 12 months.	Yes, usual care. No intervention in some.	Changes in QALY; score errors, healthcare resources, or disease avoided. No. patients on appropriate treatment/controlled/adherent/quitters	6 randomized trials + 2 cluster randomized trials + 1 multiple interrupted time series + 1 before/after
Loh ZWR (2016) [54]	5	Aug 2015	3/25 (12%)	Multicountry (1), Spain (1), Canada (1)	Total sample 3992 patients in 3 economic studies: average age approx. 75.	Medication review, patient education on drug-related problems. Follow-up 6 or 18 months.	Yes (all)	Changes in QoL. % of recommendations accepted by physician	All 3 randomized trials (inclusion criteria)
Malet-Larrea A (2016) [55]	7	Sept 2015	13/13 (100%)	Multicentric (1), UK (4), Australia (2), Canada (2), Spain (2), The Netherlands (1), Belgium (1)	Total sample 11,491 in 13 studies (median: 675): new to therapy DS patients; new to therapy elderly.	MTM (5); DSM (4); adherence/compliance (4). Median length of follow-up: 6 months.	Yes, usual care	Changes in adherence, risk/disease symptoms/severity; BP, BMI, lipids, HbA1c, PEFR; medication; use of healthcare resources; EQ-5D or other QoL; patient satisfaction	All 13 randomized trials (inclusion criteria)
Gammie T (2016) [56]	6	2010–2015	10/14 (71%)	UK (4), Spain (2), Brazil (2), France (1), Australia (1)	Total sample size NR. Patients: DS; elderly; with medication errors; at risk of apnea; smokers.	DSM (5); MTM (3); screening (1); smoking cessation (1)	Yes, usual care	Changes in adherence, disease/severity; clinical proxy outcomes; medication use; unscheduled use of healthcare resources; QoL	NR, presumably all 10 controlled

*SR* systematic review, *CP* community pharmacy, *DS* disease state, *NR* not reported, *DSM* disease state management, *MTM* medication therapy management, *CV* cardiovascular risk, *NRT* nicotine replacement therapy, *BP* blood pressure, *TG* triglycerides, *ED* emergency department, *MAI* medication appropriateness index, *CHD* coronary heart disease, *QoL* quality of life, *BMI* body mass index, *PEFR* peak expiratory flow rate

##### Economic findings of included reviews

Most reviews report medication costs, supplies/tests costs, and healthcare utilization costs. More recent reviews also report indirect costs. Very few reviews report intervention costs charged.

Only half of the reviews report cost year and discount rates of included studies. Nine reviews report the perspectives of included studies.

Almost all reviews include trial-based studies. Four reviews also report synthesis/model-based studies.

Summary measures, namely, incremental ratios, are reported in eight recent reviews. Five reviews report benefit-to-cost ratio as a summary measure.

Six reviews report uncertainty of included studies but only two of them explicit sensitivity analysis methods [24, 53].

Table 2 summarizes the major economic findings of included reviews.

**Table 2:** Economic findings of included reviews

First author (Year SR)	Types of economic evaluation in CP studies	Resource use and cost categories in CP studies	Cost year/discount rates in CP studies	Data sources in CP studies	Perspectives in CP studies	Key findings in CP studies
Schumock GY (1996) [44]	No full economic evaluation	NR	NA	Trial-based	NA	No B/C for CP studies. Cost avoided per prescription and avoided care costs per intervention
Schumock GY (2003) [45]	No full economic, 1 CCA (1)	NR (3), program, drug and healthcare costs (1), fees but lumped with drug costs savings (1), drug and advertising (1)	Not discounted (1)	Trial-based	NA	No B/C for CP studies. Lower mean total charges; lower medical and Rx costs; lower prescription costs; cost savings for interventions; costs exceed benefits (but error reported); costs per vaccination
Perez A (2008) [46]	CMA (4), CBA (2), CEA (2)	Program costs in most studies, staff time/wages/fees in some studies	Reported	Most trial-based, 1 trial/model	Reported (15)	3 B/C from CP studies (1.17; 9.47; 7.67). Decrease 57% in overall health direct and indirect costs; cost savings per patient; lower incremental cost per quitter; no significant changes in 2 studies
Chisholm Burns MA (2010) [47]	NR	Direct medical costs, Indirect costs	NR	Trial-based	NR	Improvements in HbA1c and cholesterol and decreased medical direct costs per patient per year and decreased no. of sick days every year
Touchette DR (2014) [48]	CMA (1), CUA (1)	NR	NR	6 trial-based, 1 model-based	Reported (4)	1 ICER cost-effective: 10,000£/QALY; no difference in outcomes, costs increased in 2 studies; increase in prescribing antiplatelet drug use, no cost difference at 1 year; CV medical costs decreased; direct and indirect cost savings; healthcare costs/patient/year reduced
Altowaijri A (2013) [49]	CEA (3), CUA (1), CBA (1), CMA (1)	NR	NR	5 trial-based, 2 model-based	Health system (4), society (2), not clear (1)	5 CP studies cost-effective, 1 not cost-effective (but CMA used), and 1 not full economic evaluation. Cost increase, no changes in outcomes, use of CMA questionable; cost-effective; reduction of HbA1c, cost-saving on a longer term; cost saving, gain in life years; cost-effective; program seems promising in improving patient blood pressure
Elliott RA (2014) [24]	CEA (5), CUA (3), CMA (1), CCA (6)	Patient resource use (12), costs of intervention (13), partial costs (3), costs borne by patient (1), indirect costs (2)	Reported (11)	11 trial-based, 3 model-based	Health system (12), societal (2)	This review looked at methods. 4 studies CEA, 2 CUA, 1 CEA/CUA, 1 CMA, 6 CCA. Incremental analysis used in 8 full economic evaluations: cost per error avoided, cost per extra adherent patient; cost per % increase in patient adherence; cost per quitter; cost per pelvic inflammatory disease avoided; cost per QALY
Brown TJ (2016) [50]	CEA (3), CUA (1)	Direct costs of intervention, fee charged (1), travel costs (1)	Reported	3 trial-based, 1 trial/model	Health system (3), societal (1)	All 4 economic evaluation studies reported being cost-effective ranging from 181£ to 772£ per life-year saved, ICUR 2600£; studies used ICER and 1 used ICUR.
Wang Y (2016) [51]	CEA (3), CBA (3)	Labor costs (4), cost of intervention (3), fees charged (1), transportation costs (1)	Reported	9 trial-based, 1 model-based	Payer (7), provider (3), patient (1)	Costs increased in both groups; B/C ratios favorable for 2 studies; no difference in 1 study; ICER cost-effective; costs avoided per person per year
Peletidi A (2016) [52]	CEA/CUA (1), CEA (1)	NR	NR	Trial-, model-based	NR	Both studies report incremental ratios ICER per quitter; ICUR per QALY, and cost-effective.
Perraudin C (2016) [53]	CEA (12), CUA (10), CMA (2)	Labor costs (15), costs of intervention, training costs (12), fixed costs, productivity loss OR fees charged (3)	Some: lower rates for effects	10 trial-based, 11 model-based	Payer (17); societal (5). Some both	All 21 studies are economic evaluations. ICER for most studies. CEAC ranging from 59 to 97% prob. of being C/E. Uncertainty very low for screening (chlamydia and sleep apnea) and smoking cessation. Some degree of uncertainty for remainder medication or disease interventions.
Loh ZWR (2016) [54]	CUA (1), not stated in other two.	NR	NR	All 3 trial-based	NR	1 study was 100% cost-effective when WTP threshold €30,000/QALY– €45,000/QALY; 2 studies no summary measures
Malet-Larrea A (2016) [55]	CEA/CUA (3), CUA (3), CEA (2), CCA (4), CMA (1)	Labor costs, hospital use, GP visits, medication, supplies, productivity loss	Reported for all	10 trial-based, 3 trial/model-based	Health payer (9), societal (2), govn (1), both (1). Few patient/provider	Incremental analysis performed in 9 studies and calculated for 3: 4 dominant; 7 cost-effective; 1 not cost-effective
Gammie T (2016) [56]	CUA (8), CEA (2)	NR	NR	NR	Reported (2)	ICERs performed for 9 studies: 8 are cost-effective

*SR* systematic review, *CCA* cost-consequence analysis, *CMA* cost-minimization analysis, *CBA* cost-benefit analysis, *CEA* cost-effectiveness analysis, *CUA* cost-utility analysis, *B/C* benefit-to-cost ratio, *ICER* incremental cost-effectiveness ratio, *ICUR* incremental cost-utility ratio

#### Description of community pharmacy (CP) primary studies reported in reviews

Nearly 60% of CP primary studies (index publications) are from the USA and the UK.

**Fig. 2:**
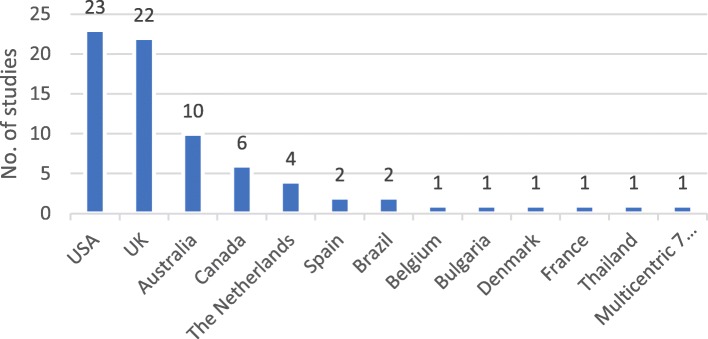
Countries of origin of economic evaluation of community pharmacy primary studies

Figure 2 illustrates countries of origin of economic evaluation of community pharmacy primary studies.

Disease management is the most frequent intervention category in primary studies (40 studies), namely, in diabetes, hypertension, hyperlipidemia, asthma, and smoking cessation, followed by medication management (13 studies) and high cost/case management, especially for the elderly on multiple medication (10 studies). Adherence is the intervention category in 5 studies for patients new to therapy, screening in 4 studies, and disease prevention (immunization) in 2 studies. The intervention for one study was not reported.

Thirty-one studies are controlled trials. Seven studies are nonrandomized trials, and one is a multiple interrupted series design. Twelve studies (16%) are model counterfactual. Twelve studies are reported as not controlled, 3 are nonconcurrent cohort studies, 2 are before/after designs, 1 is a retrospective cohort study, and no report exists for 6 studies.

**Fig. 3:**
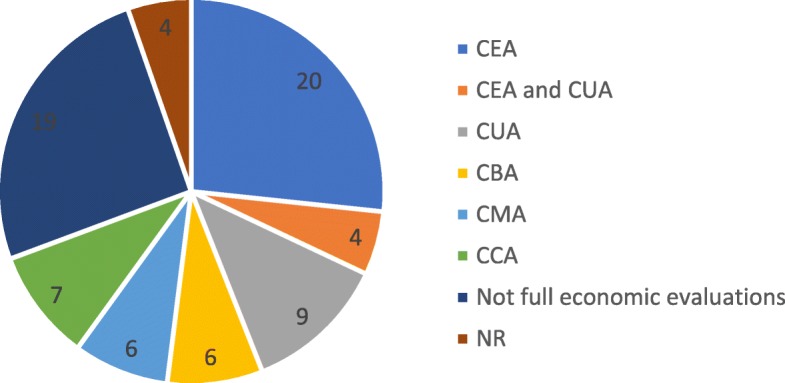
Types of economic analysis of economic evaluation CP primary studies

CEA is the most frequent type of economic analysis, followed by CUA. However, 19 studies are not full economic analyses (Fig. 3).

Health payer is the most frequent perspective reported (43 studies), societal perspective is reported for 8 studies, 2 studies seem to adopt both payer and societal perspectives, and provider perspective is reported for 6 studies. The perspective is not reported in reviews for 16 studies.

When reported, the time horizon most frequently reported is up to 12 months. Sensitivity or scenario analysis is reported for 19 studies, currency year for 50, and discount rate for 6 studies only (3%, 3.5%, and 4%), of which 2 studies present lower discount rates for consequences (1.5%). Summary measures are reported for 27 studies, 16 of which are ICERs.

Overall, reviews reported favorable economic findings for 37 of 52 studies (71%) with full economic evaluations or CCA or cost-minimization analysis (CMA).

See supplementary characteristics of primary studies as reported in included reviews, Additional file 4.

#### Methodological quality of included reviews

##### Quality of included reviews

In accordance with recommended approach in AMSTAR 2, we identified (four) critical domains; we assessed all 16 items for each included review but did not use responses to derive an overall score; and we rated overall confidence on the four critical domains as high (green), moderate (yellow), or low (red). The two critical quality domains that seem to be absent from almost all reviews are protocol registered before commencement of the review and no justification for excluding individual studies.

Table 3 summarizes the results of quality assessment of included reviews in the considered four critical domains of AMSTAR 2.

**Table 3:** Quality of included reviews in critical domains (AMSTAR 2)

No.	AMSTAR 2 item	*n* (%)	Critical domains
2	Explicit statement—review methods established prior to review and justification for deviations from protocol	2 (14%)	
4	Used comprehensive literature search strategy	14 (100%)	
7	Provided list of excluded studies and justification for exclusions	0 (0%)	
13	Accounted for risk of bias in individual studies when interpreting/discussing results of review	10 (71%)	

See supplementary complete quality assessment of included reviews in all 16 items of AMSTAR, Additional file 5.

##### Quality of evidence of CP primary studies reported in reviews

We assessed the quality of evidence of CP primary studies reported in Reviews in five areas: quality of evidence for deriving effectiveness; risk of bias; quality of economic evaluation; heterogeneity; wider research issues.

***Quality of evidence of CP primary studies for deriving effectiveness***. Three reviews defined RCTs as one inclusion criteria [49, 54, 55]. Three others used an instrument to assess the quality of included study designs [24, 47, 50]. Chisholm-Burns et al. used a simple hierarchy of study designs, Elliot et al. used Standard Hierarchies of Evidence [57], and Brown et al. used Cochrane’s Effective Practice and Organization of Care (EPOC) study design criteria [40]. Thirty-nine of 57 reported trial-based studies (68%) used an EPOC review recommended study design.

***Risk of bias of CP primary studies***. Three recent reviews used an instrument to assess the risk of bias of included studies [50, 54, 55].

Brown et al. used the six-item Effective Public Health Practice Project Quality Assessment Tool for Quantitative Studies [58]. This review included randomized and nonrandomized trials. Six studies were rated “strong,” four studies were rated “moderate,” and nine studies were rated “weak.”

Loh et al. used the seven-item Cochrane Collaboration’s tool for assessing risk of bias in randomized trials [59]. Selection bias (random allocation and allocation concealment) was the highest risk rated. However, low risk of bias represented more than 50% of items scored in all studies except one.

Malet-Larrea et al. used the nine-item Risk of Bias for EPOC Reviews Tool [41]. This review included randomized trials only. Seven studies were rated high risk, four were rated medium risk, and two were rated low risk. Risk of contamination and not reporting baseline characteristics of providers were the highest risks rated.

Six reviews used no instrument but reported risk of bias in included studies [24, 45, 46, 48, 51, 53]. Assisted by the Risk of Bias for EPOC Reviews Tool, we identified random sequence generation, allocation concealment, baseline characteristics, baseline outcome measurements, and protection against contamination as the most frequent items reported for higher risk of bias in these reviews.

One review assessed but reported no significant bias [56]. Four reviews did not assess risk of bias [44, 47, 49, 52].

***Quality of economic evaluation of CP primary studies***. Five reviews used an instrument to assess the quality of economic evaluations of included studies [24, 46, 48, 51, 55].

Perez et al. used an instrument developed by the authors [46]. Touchette et al. used the Quality of Health Economic Studies (QHES) for the total 18 full economic evaluations [48]. The first review to use CHEERS was Elliot’s [24]. Worst performance criteria seem to be the following: contamination between intervention and comparator, costs incurred by patient, indirect costs, discounting, accounting for uncertainty, summary measures, and sensitivity or scenario analyses.

Wang et al. also used CHEERS [51]. The authors reported that many of the studies met most CHEERS criteria despite some issues in presenting summary measures, in dealing with uncertainty and with impact of heterogeneity.

Malet-Larrea et al. used the Evers Checklist Tool used for all economic evaluations in addition to the Phillips instrument for three combined trial and model-based economic evaluations [55]. The authors also reported a high quality in eight studies, medium quality in three studies, and low quality in two studies.

Six other reviews did not use an instrument but performed some assessment of economic evaluation in the included studies [44, 45, 47, 49, 53, 56]. Assisted by CHEERS, we identified criteria that present some issues based on reported data of primary studies in reviews: not detailing characteristics of target population; not describing “usual care” comparators; not including patient costs, indirect costs or intervention fees; analytical methods poorly described; incremental costs and outcomes sometimes not reported; not accounting for uncertainty; issues in characterizing heterogeneity; and not reporting the source of funding of studies.

***Heterogeneity (populations, interventions, outcomes) of CP primary studies***. Ten reviews include studies addressing diverse populations, interventions, outcomes, and even settings, and most report this variability, which does not allow synthesizing results or generalizing [24, 44–46, 48–51, 53, 55].

Four reviews do not assess or report the impact of heterogeneity in results [47, 52, 54, 56].

***Wider research issues of CP primary studies***. Twelve reviews do not report equity considerations—e.g., whether socioeconomic variables had differential effect on intervention—an important aspect in public health interventions [24, 44–46, 48, 49, 51–56].

Nine reviews provide some meager considerations on the sustainability of interventions [24, 45, 46, 48, 50, 52, 53, 55, 56]. However, most of these are restricted to training of staff and integration into routine and provide no information on the economic and political variables to understand its impact on sustainability. Five reviews do not report any assessment of sustainability [44, 47, 49, 51, 54].

Eight reviews provide some considerations on the context of the included studies [46, 48, 50–53, 55, 56]. US reviews focus on framework legislation encouraging added value interventions while European reviews tend to address context barriers, including lack of funding for cost-effective interventions. Six reviews, however, do not address the context of included studies [24, 44, 45, 47, 49, 54].

##### Applicability and transferability

Assisted by Wang’s framework, nine reviews briefly discuss the importance of process dimensions for applicability: political environment, resource implications, and organizational structure of pharmacies and skill of pharmacists. The social acceptability by the target population, cultural adaptability, and the impact of the educational level of the target population are not reported in reviews.

Reviews do not seem to discuss the importance of the following outcome dimensions for transferability: baseline prevalence of the condition, differences in populations, and capacity to implement the intervention. However, despite variations in populations and in the capacity to implement the intervention, we observe some consistency in favorable findings for some intervention categories across different countries.

#### Synthesis of results

Economic evaluations of pharmacy-based public health interventions include various elements, additional dimensions, and challenges which are summarized in Table 4.

**Table 4:** Major key methodological findings

Most frequent risk of biases	Random sequence generation, allocation concealment, baseline characteristics, baseline outcome measurements similar, and contamination between intervention and comparator
Study designs	Not restricted to RCTs or cluster RCTs
Economic quality criteria	Most criteria are met but there are some issues: not detailing target population; not describing “usual care” comparators; not including patient costs, indirect costs or intervention fees; analytical methods poorly described; incremental costs and outcomes sometimes not reported; not accounting for uncertainty
Heterogeneity	In populations, interventions and some outcomes
Equity	Not assessed
Process dimensions	Process dimensions that impact dissemination and external validity poorly described.

*RCT* randomized controlled trial

Favorable economic findings appear for 71% of studies with full economic evaluations or CCA or CMA.

In triangulation with recommendations for economic evaluations of both public health interventions and alongside trials, we assessed all ten items of these recommendations for each included review and we rated collective overall agreement of reviews with each item as high (green), moderate (yellow), or low (red).

Triangulation with recommendations for economic evaluations both of public health interventions and alongside trials reveals poor evidence of the following: societal perspective, costs and benefits falling on nonhealth sectors, lower discounting rates (vs medicines), equity assessment, valuation of costs, and methods for costs and outcome analysis.

The following recommendations are present but not well detailed: prior review of evidence, linking intermediate to long-term outcomes, wider spectrum of research methods, selection of subjects and sites seeking real-world target population and providers, current practice or standard of care, cost data collection, and detailed reporting.

In contrast, the following recommendations are usually present and well detailed: economic appraisal is linked to effectiveness, most study designs are RCTs but include other controlled designs, the types of economic evaluation are diverse, study designs tend to be pragmatic, sample size is based on an important clinical outcome correlated with economic outcomes, and single direct measures are usually preferred.

Tables 5 and 6 summarize the triangulation with recommendations.

**Table 5:** Triangulation with key recommendations on the economic evaluation of public health interventions

#	Key recommendations	Overview findings
1	Review of evidence: Systematic review or in-depth review of evidence prior to the economic evaluation of PHI	
2	Effectiveness and economic appraisal: Economic appraisal linked to the appraisal of effectiveness of PHI	
3	Study designs: When randomized trial not feasible, quasi-experimental designs or econometric techniques	
4	Perspective: Societal perspective (public sector may be used where appropriate)	
5	Time horizon: Trial data may need modeling but requires reliable link between intermediate and long-term outcomes	
6	Types of economic evaluation preferred: CBA and CCA preferred but CUA and CEA also recommended whenever health is the sole benefit	
7	Nonhealth costs and benefits of PHI: Need to capture costs and benefits falling on nonhealth sectors	
8	Discounting rates: Lower discounting rates for PHI (1.5% for NICE, 3% for CDC) if costs and health effects accrued > 1 year	
9	Equity considerations: Compare differences in health status changes between different health economic groups	
10	Wider spectrum of research methods: Understand contextual and process indicators affecting behavior change and other variables	

*PHI* public health interventions, *CBA* cost-benefit analysis, *CCA* cost-consequence analysis, *CUA* cost-utility analysis, *CEA* cost-effectiveness analysis, *NICE* National Institute for Health and Care Excellence, *CDC* Centers for Disease Control and Prevention

**Table 6:** Triangulation with key recommendations on the economic evaluation alongside clinical or pragmatic trials

#	Key recommendations	Overview findings
1	Study design: Based on well-designed pragmatic/naturalistic trials with fewer strict protocols	
2	Selection of subjects and sites: Seek for proximity to real-world target population and less restrictive patient inclusion criteria	
3	Sample size: Based on important clinical outcome correlated with economic outcome, previous pilot or wider CI for ICER/CEAC	
4	Estimates beyond trial: Appropriate length of follow-up, estimates beyond trial require survival analysis, link to final outcomes or regression	
5	Comparator: Current practice or standard of care should be the comparator, although there may be different standards of care	
6	Measures of outcomes: Direct, single measures are preferred. Utilities collected directly from study subjects at regular intervals	
7	Data collection (resource use and costs): Relevant resource use and cost measures collected with clinical data (case report forms, patient records, patient diaries, interviews, computerized record linkage)	
8	Valuation of costs: May include: microcosting; unit costing; and gross costing	
9	Methods for cost and outcome analysis: Arithmetic mean cost differences, bootstrapping, OLS or GLM for between group comparison; multivariable methods for outcomes; confidence intervals, *p* values, ICERs on various time horizons; summary measures	
10	Reporting: General description of trial and major findings; economic data collected alongside trial; missing and censored data; methods to construct, compare and project costs and outcomes; statistical methods; results on resource use, costs and outcomes; results within and beyond time horizon of trial.	

*CI* confidence intervals, *ICER* incremental cost-effectiveness ratio, *CEAC* cost-effectiveness acceptability curve

## Discussion

### Summary of evidence

Economic evaluations of pharmacy-based public health interventions seem to follow most economic quality criteria, but there are still some issues in certain key areas to improve.

This overview sought to include all relevant systematic reviews complying with inclusion criteria, with no restrictions on the period covered, populations, interventions, comparators, or outcomes, in attempt to be inclusive and to critique methods.

We have defined four of seven AMSTAR 2 critical domains for an overall critique on how well included reviews performed in these domains, as recommended. Risk of bias from individual studies being included in the review was not considered, since the most important instruments for nonrandomized studies included in a systematic review were released in 2016 and 2017. Appropriateness of meta-analytical methods was not considered, since included reviews were narrative. Assessment of presence and likely impact of publication bias was also not considered, since funnel plot asymmetry requires a minimum of ten studies and included reviews each have eight studies on average.

The findings of this overview are consistent with methodological issues described by Elliot et al., Perraudin et al., Malet-Larrea et al., Jommy, and Whitty for the economic evaluation of pharmacy-based interventions [24, 53, 55, 60, 61]. In addition, they are also consistent with the major methodological challenges described by Weatherly et al. for the economic evaluation of public health interventions [15] and with the methodological challenges described by O’Sullivan et al. for the economic evaluation conducted alongside clinical trials [17].

### Limitations

This review only included those studies containing the selected search terms. Hence, it is possible we might have missed relevant systematic reviews that may have used a different terminology.

We used a specific filter recommended by the Centre for Reviews and Dissemination (CRD) for economic evaluation in Embase via Ovid, as stated in Additional file 2.

We did not find a specific filter for economic evaluation for other databases used in this review published in ISSG Search Filter Resource. We restricted the search strategy to the definition of full economic evaluation, that is, comparing costs and health outcomes of two or more interventions in which three generic types of economic evaluation are used: cost-effectiveness, cost-utility, or cost-benefit analyses. We deliberately excluded search terms used in partial economic evaluations. However, this restriction might have implicated on the sensitivity of these searches.

In addition, it is possible we might have missed relevant recommendations on economic evaluations of public health interventions and/or alongside trials due to the selected exploratory approach used for these reviews with the sole purpose of obtaining key insights for triangulation.

## Conclusions

### Implications for practice and policy

In recent years, governments in various countries have introduced profound changes to pharmacy remuneration systems in the components related to equitable, safe, and quality access to medicines and to efficiency and/or quality incentives. These two components are the most important in pharmacy remuneration systems. However, the network of pharmacies provides a unique opportunity for governments to implement relevant public health interventions, that fit within national, regional and local health policies, in close collaboration with primary care. In several countries, governments have already contracted with pharmacies to pay for relevant interventions. However, as for any other health technology, public health interventions provided by community pharmacists must seek to demonstrate effectiveness and economic benefits to be reimbursed by public payers.

The economic evaluation of pharmacy interventions presents challenges.

We hope the findings of this overview may assist in improving the design, implementation, and assessment of pilot trials; hence, the robustness of evidence to justify payers’ investment, which requires the endorsement of community pharmacists to participate in trials and an informed understanding of policy makers in negotiations.

### Implications for research

Based on the findings of this overview and in addition to the methodological considerations for economic evaluations of pharmacist interventions by Elliot et al. [24], we propose a methodological approach for the economic evaluation of pharmacy-based public health interventions (see Table 7).

**Table 7:** Methodological approach for the economic evaluation of pharmacy-based public health interventions

Step	Methodological approach	
1	Performing a systematic or in-depth review of existing evidence prior to economic evaluation	
2	Planning and conducting well-designed trial(s) for the assessment of effectiveness using	PICO framework for a clear definition of population, intervention, comparator and outcomes
		EPOC study design to assist in the selection of the best possible study design
		Risk of Bias for EPOC Reviews Tool to assist in strategies to minimize the most frequent risk of bias
3	Planning and conducting an economic evaluation using:	CHEERS Checklist to perform economic evaluations according to accepted standards
		Recommendations for economic evaluations of public health interventions and for economic evaluations alongside trials to assist in adjustments: using a societal perspective; reliable linking of intermediate to long-term outcomes; choice of health outcomes may not allow for QALY; if costs and benefits also fall on nonhealth sectors, CBA approach may also be required; intervention costs must consider a retail price (as this would be the case if reimbursed); use of lower discount rates; other summary measures may be required if CEA nor CUA are used; and performing equity assessment
4	Using a wider spectrum of research methods to understand:	Contextual and process indicators affecting the behavior change of patients and providers and other variables

*PICO* population, intervention, comparator, outcomes, *EPOC* Effective Practice and Organization of Care, *CHEERS* Consolidating Health Economic Evaluation Reporting Standards, *QALY* quality-adjusted life years, *CBA* cost-benefit analysis, *CEA* cost-effectiveness analysis, *CUA* cost-utility analysis

As the research corpus continues to expand following practice and policy requirements, it will become important to build a multidisciplinary expert consensus around a specific guidance for the economic evaluation of pharmacy-based public health interventions.

## Supplementary information

**Additional file 1.** Completed PRISMA Checklist.pdf (Completed PRISMA Checklist) – this file contains the completed PRISMA items checklist.

**Additional file 2.** Search Strategies.pdf (Search strategies) – this file contains the search terms and search strategies used for electronic databases.

**Additional file 3.** List of Excluded Reviews.pdf (List of excluded reviews) – this file contains the list of excluded reviews during the study selection process.

**Additional file 4.** Characteristics of Primary Studies.xls (Characteristics of primary studies as reported in included reviews) – this file contains a table of all 75 primary studies and its main characteristics as reported in included reviews.

**Additional file 5.** Quality of included reviews in all 16 items (AMSTAR 2) – this file contains the complete quality assessment of included reviews in all 16 items of AMSTAR 2.

## Data Availability

All data generated or analyzed during this research are included in this published article. Additional supporting information may be found in the following: • Completed PRISMA Checklist (Additional file 1) • Search strategies (Additional file 2) • List of excluded reviews (Additional file 3) • Characteristics of primary studies as reported in included reviews (Additional file 4) • Quality of included reviews in all 16 items (AMSTAR 2) (Additional file 5).

## Abbreviations

AMSTAR-2: Assessing the Methodological Quality of Systematic Reviews
CBA: Cost-benefit analysis
CCA: Corrected covered area
CCA: Cost-consequence analysis
CDC: Centers for Disease Control and Prevention
CDSR: Cochrane Database of Systematic Reviews
CEA: Cost-effectiveness analysis
CHEERS: Consolidated Health Economic Evaluation Reporting Standards
CMA: Cost-minimization analysis
CP: Community pharmacy
CRD: Centre for Reviews and Dissemination
CRF: Case report forms
CUA: Cost-utility analysis
DARE: Database of Abstracts of Reviews of Effects
EPOC: Effective Practice and Organization of Care
EU: European Union
GLM: Generalized linear model
ICER: Incremental cost-effectiveness ratio
ISPOR: International Society for Pharmacoeconomics and Outcomes Research
JBI: Joanna Briggs Institute
MCDA: Multiple-criteria decision analysis
MRC: Medical Research Council
NHS EED: National Health Service Economic Evaluation Database
NICE: The National Institute for Health and Care Excellence
OECD: Organization for Economic Co-operation and Development
OLS: Ordinary least squares
PRISMA: Preferred Reporting Items for Systematic Reviews and Meta-Analyses
PROSPERO: International Prospective Register of Systematic Reviews
QALY: Quality-adjusted life years
QHES: Quality of Health Economic Studies
RCT: Randomized controlled trials
