# Supplementary material for: An overview of systematic reviews of economic evaluations of pharmacy-based public health interventions: addressing methodological challenges
Source: Syst Rev. 2019 Nov 11;8:272. doi: 10.1186/s13643-019-1177-3 (PMC6844055; doi:10.1186/s13643-019-1177-3)
Supplement: Supplementary file 2 — Additional file 2. Search Strategies.pdf (Search strategies) – this file contains the search terms and search strategies used for electronic databases. [file 13643_2019_1177_MOESM2_ESM.pdf]

An Overview of Systematic Reviews of Economic Evaluations of Pharmacy-Based Public Health Interventions: Addressing Methodological Challenges

Additional File 2: Search Strategies

All searches performed until July 2017.

Embase & MEDLINE (via Ovid)

Database: Embase <1996 to 2017 Week 29>, Epub Ahead of Print, In-Process & Other Non-Indexed Citations, Ovid MEDLINE(R) Daily and Ovid MEDLINE(R) <1946 to Present>

We used filter Centre for Reviews and Dissemination NHS EED Embase using OvidSP for economic evaluation (except we have not restricted to inception date 2010) and filter Centre for Reviews and Dissemination (Strategy 2.2) MEDLINE using OvidSP for systematic reviews, as detailed below.

Search Strategy:
----
1 (((pharmacy or pharmacist) adj2 Intervention$) or ((pharmacy or pharmacist) adj2 service$) or ((pharmacy or pharmacist) adj2 program$) or ((pharmacy or pharmacist) adj2 manage$)).ti,ab. (14283)
2 Health Economics/ (18081)
3 exp Economic Evaluation/ (281586)
4 exp Health Care Cost/ (261336)
5 pharmacoeconomics/ (8678)
6 2 or 3 or 4 or 5 (481060)
7 (econom$ or cost or costs or costly or costing or price or prices or pricing or pharmacoeconomic$).ti,ab. (1226685)
8 (expenditure$ not energy).ti,ab. (46224)
9 (value adj2 money).ti,ab. (2831)
10 budget$.ti,ab. (45097)
11 7 or 8 or 9 or 10 (1271562)
12 6 or 11 (1462701)
13 letter.pt. (1599098)
14 editorial.pt. (846451)
15 note.pt. (549945)
16 13 or 14 or 15 (2995412)
17 12 not 16 (1369782)
18 (metabolic adj cost).ti,ab. (1948)
19 ((energy or oxygen) adj cost).ti,ab. (5787)
20 ((energy or oxygen) adj expenditure).ti,ab. (40697)
21 18 or 19 or 20 (46979)
22 17 not 21 (1359971)
23 animal/ (6805335)
24 exp animal experiment/ (1137690)
25 nonhuman/ (3439045)
26 (rat or rats or mouse or mice or hamster or hamsters or animal or animals or dog or dogs or cat or cats or bovine or sheep).ti,ab,sh. (9455604)

27 23 or 24 or 25 or 26 (11167614)
28 exp human/ (28601606)
29 human experiment/ (226677)
30 28 or 29 (28601872)
31 27 not (27 and 30) {No Related Terms} (95174)
32 22 not 31 (1355816)
33 meta-analysis/ (182870)
34 review literature/ (2210684)
35 meta-analy$.tw. (222614)
36 metaanal$.tw. (7838)
37 (systematic$ adj4 (review$ or overview$)).mp. (272446)
38 meta-analysis.pt. (72900)
39 review.pt. (3926614)
40 review.ti. (619872)
41 review literature.pt. (0)
42 or/33-41 (4407018)
43 case report/ (3107267)
44 historical article.pt. (336038)
45 review of reported cases.pt. (0)
46 review,multicase.pt. (0)
47 or/43-46 (3441428)
48 42 not 47 (4174558)
49 1 and 32 and 48 (614)
50 remove duplicates from 49 (508)

DARE, NHS EED, HTA

(systematic review OR meta-analysis) AND (economic evaluation OR (economic OR cost-effectiveness OR cost-benefit OR cost-utility)) AND (((pharmacy OR pharmacist) ADJ2 intervention*) OR ((pharmacy OR pharmacist) ADJ2 service*) OR ((pharmacy OR pharmacist) ADJ2 program*) OR ((pharmacy OR pharmacist) ADJ2 manage*))

Tufts CEA Registry

1st search: pharmacy
2nd search: pharmacist
OBS: Basic search in this database (in free access mode) allows for one word only and it is not possible to use Boolean operators, hence requiring two separate searches and selecting the most specific term(s).

Cochrane Database of Systematic Reviews (CDSR)

#1 "systematic review" or "meta-analysis"
#2 "economic evaluation" or ("economic" or "cost-effectiveness" or "cost-benefit" or "cost-utility")
#3 (("pharmacy" or "pharmacist") near/2 intervention) or (("pharmacy" or "pharmacist") near/2 service) or (("pharmacy" or "pharmacist") near/2 program) or (("pharmacy" or "pharmacist") near/2 (managed or management))

#4 #1 and #2 and #3

Web of Science (WoS)

("systematic review" OR meta-analysis) [title] AND ("economic evaluation" OR economic OR cost-effectiveness OR cost-benefit OR cost-utility) [topic] AND (((pharmacy OR pharmacist) NEAR/2 intervention*) OR ((pharmacy OR pharmacist) NEAR/2 service*) OR ((pharmacy OR pharmacist) NEAR/2 program*) OR ((pharmacy OR pharmacist) NEAR/2 manage*)) [topic]

Google Scholar

Search performed under "Advanced Search", limited to articles, where words occur anywhere in title of the article, returning articles dated from 2016 onwards:

With all of the words: "pharmacist"
With the exact phrase: "systematic review"
With at least one of the words: "economic evaluation" "economic" "cost effectiveness" "cost benefit" "cost utility"
Without the words: "hospital" "pharmacological"

PROSPERO Registry

Search performed to identify possible ongoing, ongoing update or completed (but not published neither abandoned) systematic reviews:

("systematic review" OR meta-analysis) AND ("economic evaluation" OR economic OR "cost-effectiveness" OR "cost-benefit" OR "cost-utility") AND ((pharmacy OR pharmacist) ADJ2 (intervention* OR service* OR program* OR manage*)) AND (ongoing OR completed OR ongoing_update):RS

Grey Literature – ISPOR Database

Search for pharmacy or pharmacist (filter for Cost studies to capture economic evaluation studies).

Grey Literature – OpenGrey Database

("systematic review" or meta-analysis) AND economic AND pharmacy (filter: discipline: (05T – Health services, health administration, community care services)
