# Supplementary material for: An overview of systematic reviews of economic evaluations of pharmacy-based public health interventions: addressing methodological challenges
Source: Syst Rev. 2019 Nov 11;8:272. doi: 10.1186/s13643-019-1177-3 (PMC6844055; doi:10.1186/s13643-019-1177-3)
Supplement: Supplementary file 3 — Additional file 3. List of Excluded Reviews.pdf (List of excluded reviews) – this file contains the list of excluded reviews during the study selection process. [file 13643_2019_1177_MOESM3_ESM.pdf]

An Overview of Systematic Reviews of Economic Evaluations of Pharmacy-Based Public Health Interventions: Addressing Methodological Challenges

Additional File 3: List of excluded reviews (n=702, after removing 45 initial duplicates)

ID	Database	Title
#1	MEDLINE_EMBASE	Pharmaceutical services as a tool to improve outcomes in patients with cardiovascular diseases.
#2	MEDLINE_EMBASE	A systematic review of evidence-based community pharmacy services aimed at the prevention of cardiovascular disease.
#3	MEDLINE_EMBASE	Effectiveness of pharmaceutical care in medicated hypertensive patients: AFPRES CLM Study.
#4	MEDLINE_EMBASE	Pharmacist-led interventions to reduce unplanned admissions for older people: A systematic review and meta-analysis of randomised controlled trials.
#5	MEDLINE_EMBASE	Specialty pharmacy services: Preparing for a New Era in health-system pharmacy.
#6	MEDLINE_EMBASE	Cluster randomized trials for pharmacy practice research.
#7	MEDLINE_EMBASE	Economic model to examine the cost benefit associated with resolution or improvement of carcinoid syndrome symptoms following treatment with above-standard dose of octreotidelar in patients with neuroendocrine tumors based on data from a retrospective chart review study at three large tertiary oncology centers in the United States.
#8	MEDLINE_EMBASE	44th ESCP International Symposium on Clinical Pharmacy Medicines Information: Making Better Decisions.
#9	MEDLINE_EMBASE	Identification of indicators for evaluating the financial and economic performance of the pharmacy: A systematic review.
#10	MEDLINE_EMBASE	Cost-avoidance and qualitative analysis of clinical pharmacy interventions by psychiatric pharmacy residents at state psychiatric facilities.
#11	MEDLINE_EMBASE	Pharmacist-involved care for patients with heart failure and acute coronary syndrome: A systematic review with qualitative and quantitative meta-analysis.
#14	MEDLINE_EMBASE	The Role of the Clinical Pharmacist in the Care of Patients with Cardiovascular Disease.
#15	MEDLINE_EMBASE	Comparative efficacy and safety of estradiol transdermal preparations for the treatment of vasomotor symptoms in postmenopausal women: An indirect comparison meta-analysis.
#16	MEDLINE_EMBASE	Medication adherence to oral cancer therapy: The promising role of the pharmacist.
#17	MEDLINE_EMBASE	Hospital pharmacist-led review of medication in the home in the paediatric palliative care population.
#18	MEDLINE_EMBASE	Tools for Assessing Potential Significance of Pharmacist Interventions: A Systematic Review.
#19	MEDLINE_EMBASE	FDAMA Section 114: Why the Renewed Interest?.
#20	MEDLINE_EMBASE	A systematic review on comparing 2 common models for management of warfarin therapy; Pharmacist-led service versus usual medical care.
#21	MEDLINE_EMBASE	Pharmacist-managed clinics for patient education and counseling in Japan: Current status and future perspectives.
#22	MEDLINE_EMBASE	Pharmacy care and adherence to primary and secondary prevention cardiovascular medication: A systematic review of studies.
#23	MEDLINE_EMBASE	Effectiveness of practices to increase timeliness of providing targeted therapy for inpatients with bloodstream infections: A laboratory medicine best practices systematic review and meta-analysis.
#24	MEDLINE_EMBASE	Clinical pharmacy service practice in a Chinese tertiary hospital.
#25	MEDLINE_EMBASE	Medicine non-adherence in kidney transplantation.
#26	MEDLINE_EMBASE	Standardized patients in pharmacy education: An integrative literature review.
#28	MEDLINE_EMBASE	Pharmacists performing quality spirometry testing: an evidence based review.
#29	MEDLINE_EMBASE	Implementation of medication review with follow-up in a Spanish community pharmacy and its achieved outcomes.
#30	MEDLINE_EMBASE	Literature review: pharmacists' interventions to improve control and management in type 2 diabetes mellitus.
#31	MEDLINE_EMBASE	Strategic considerations for centralization of services across the pharmacy enterprise.
#32	MEDLINE_EMBASE	Expanding clinical and information services to the ambulatory older adult through community outreach programs.
#33	MEDLINE_EMBASE	Physician-pharmacist collaborative care model for buprenorphine-maintained opioid-dependent patients.
#34	MEDLINE_EMBASE	Three-year review of pharmacy students' interventions and activities in an outpatient teaching family medicine center.
#35	MEDLINE_EMBASE	Medication therapy management interventions in outpatient settings: A systematic review and meta-analysis.
#36	MEDLINE_EMBASE	Community pharmacy interventions for public health priorities: A systematic review of community pharmacy-delivered weight, smoking and alcohol management interventions.
#37	MEDLINE_EMBASE	Overview of systematic reviews assessing the impact of clinical pharmacy services.
#38	MEDLINE_EMBASE	Development of a multidimensional scale "CLEO" for evaluation of clinical, economic, and organizational impacts of a pharmacist intervention.
#39	MEDLINE_EMBASE	A comprehensive review of the impact of clinical pharmacy services on patient outcomes in mental health.
#40	MEDLINE_EMBASE	Improving blood pressure control through pharmacist interventions: a meta-analysis of randomized controlled trials.

#41	MEDLINE_EMBASE	Integrated clinical and specialty pharmacy practice model for management of patients with multiple sclerosis.
#42	MEDLINE_EMBASE	New anticancer agents: role of clinical pharmacy services.
#43	MEDLINE_EMBASE	On-call pharmacist enquiries and outcomes: An on-call database review, 2003-2012.
#44	MEDLINE_EMBASE	Community pharmacy interventions for public health priorities: Protocol for a systematic review of community pharmacy-delivered smoking, alcohol and weight management interventions.
#45	MEDLINE_EMBASE	Economic evaluations of clinical pharmacist interventions on hospital inpatients: a systematic review of recent literature.
#46	MEDLINE_EMBASE	Evaluation of pharmacist care for patients with chronic obstructive pulmonary disease: a systematic review and meta-analysis.
#47	MEDLINE_EMBASE	Provision of clinical pharmacist services for individuals with chronic hepatitis C viral infection: Joint opinion of the GI/liver/nutrition and infectious diseases practice and research networks of the American College of Clinical Pharmacy.
#50	MEDLINE_EMBASE	The survey of patients' satisfaction with outpatient pharmacy and systematic review of dispensing mode in mainland China. [Chinese]
#51	MEDLINE_EMBASE	The pharmacist role in predicting and improving medication adherence in heart failure patients.
#52	MEDLINE_EMBASE	A systematic literature review assessing the directional impact of managed care formulary restrictions on medication adherence, clinical outcomes, economic outcomes, and health care resource utilization.
#53	MEDLINE_EMBASE	Balanced scorecards as a tool for developing patient-centered pharmacy services.
#54	MEDLINE_EMBASE	The impact of 5-HT3RA use on cost and utilization in patients with chemotherapy-induced nausea and vomiting: Systematic review of the literature.
#55	MEDLINE_EMBASE	The significance of pharmacy interventions: An updated review in the presence of electronic order entry.
#56	MEDLINE_EMBASE	Issues facing pharmacy leaders in 2014: Suggestions for pharmacy strategic planning.
#57	MEDLINE_EMBASE	Remember the MaineRx.
#58	MEDLINE_EMBASE	Economic burden of urgency urinary incontinence in the United States: A systematic review.
#59	MEDLINE_EMBASE	Pharmacists' role in addressing opioid abuse, addiction, and diversion.
#60	MEDLINE_EMBASE	A systematic review and meta-analysis of pharmacist-led fee-for-services medication review.
#61	MEDLINE_EMBASE	The grace checklist for rating the quality of observational studies of comparative effectiveness: A tale of hope and caution.
#62	MEDLINE_EMBASE	Development and validation of a multidimensional scale "CLEO" for evaluating potential significance of pharmacist interventions.
#63	MEDLINE_EMBASE	"Healthcare Cost, Quality, and Policy: Driving Stakeholder Innovation in Process and Practice".
#64	MEDLINE_EMBASE	Review on medicines prices and availability in Indonesia: 2004 to 2012.
#65	MEDLINE_EMBASE	Pharmacotherapy review as a safety and cost tool in patients management in Slovenian Psychiatric Hospital.
#66	MEDLINE_EMBASE	Can community pharmacy contribute to weight management? A qualitative study of the perspectives of the pharmacy team.
#67	MEDLINE_EMBASE	APhA2014 Abstracts.
#68	MEDLINE_EMBASE	Pharmacist vaccination programs for common infectious diseases: A systematic review of the literature on this emerging model of care.
#69	MEDLINE_EMBASE	Quality of pharmacist-managed anticoagulation therapy in long-term ambulatory settings: A systematic review.
#70	MEDLINE_EMBASE	The impact of intra-operative dexmedetomidine infusion on immediate postoperative pain: A systematic review protocol.
#71	MEDLINE_EMBASE	Consortium recommendations for advancing pharmacists' patient care services and collaborative practice agreements.
#72	MEDLINE_EMBASE	Assessing the Implementability of Clinical Pharmacist Interventions in Patients With Chronic Kidney Disease: An Analysis of Systematic Reviews.
#73	MEDLINE_EMBASE	Pharmacist contributions as members of the multidisciplinary ICU team.
#74	MEDLINE_EMBASE	A systematic review of the economic and humanistic burden of illness in irritable bowel syndrome and chronic constipation.
#75	MEDLINE_EMBASE	A survey of individuals in US-based pharmaceutical industry HEOR departments: Attitudes on policy topics.
#76	MEDLINE_EMBASE	New models of chronic kidney disease care including pharmacists: Improving medication reconciliation and medication management.
#77	MEDLINE_EMBASE	Medication underdosing and underprescribing: Issues that may contribute to polypharmacy, poor outcomes.
#78	MEDLINE_EMBASE	The effect of pharmacist-provided non-dispensing services on patient outcomes, health service utilisation and costs in low- and middle-income countries.
#79	MEDLINE_EMBASE	Clinical pharmacy services in heart failure: An opinion paper from the heart failure Society of America and American College of Clinical Pharmacy Cardiology Practice and Research Network.
#80	MEDLINE_EMBASE	Expanding the role of community pharmacists: Policymaking in the absence of policy-relevant evidence?.
#82	MEDLINE_EMBASE	Medication Reviews by Clinical Pharmacists at Hospitals Lead to Improved Patient Outcomes: A Systematic Review.
#83	MEDLINE_EMBASE	Issues facing pharmacy leaders in 2013.
#84	MEDLINE_EMBASE	Transforming hypertension management using mobile health technology for telemonitoring and self-care support.

#85	MEDLINE_EMBASE	Implications of real-world adherence on cost-effectiveness analysis in multiple sclerosis.
#86	MEDLINE_EMBASE	Narrative review of the literature on adherence to disease- modifying therapies among patients with multiple sclerosis.
#87	MEDLINE_EMBASE	Patient perceptions of the value of pharmacy-based clinical services: A systematic review.
#88	MEDLINE_EMBASE	A systematic review of community pharmacy-based interventions for smoking cessation.
#89	MEDLINE_EMBASE	A systematic literature review of the impact of 5-HT3RA use on health care utilization in patients with chemotherapy-induced nausea and vomiting in the United States.
#90	MEDLINE_EMBASE	Methods assessing the significance of a pharmacist intervention: Literature review.
#91	MEDLINE_EMBASE	Biosimilar medicines in the management of the hospital pharmacy.
#92	MEDLINE_EMBASE	Colloque Annuel 2013 du Regroupement Quebecois de Recherche sur L'Usage du Medicament. [French, English]
#93	MEDLINE_EMBASE	CPNP's 16th Annual Meeting.
#94	MEDLINE_EMBASE	Improving inappropriate medication use among elderly veterans: Impact of medication review on polypharmacy.
#95	MEDLINE_EMBASE	The potential role of clinical pharmacy services in patients with cardiovascular diseases.
#96	MEDLINE_EMBASE	Review of survey articles regarding medication therapy management (MTM) services/programs in the United States.
#97	MEDLINE_EMBASE	Effect of a pharmacist-managed hypertension program on health system costs: An evaluation of the study of cardiovascular risk intervention by pharmacists-hypertension (SCRIP-HTN).
#98	MEDLINE_EMBASE	Implementation of a comprehensive pharmaceutical care program for an underserved population.
#99	MEDLINE_EMBASE	An analysis of quality of systematic reviews on pharmacist health interventions.
#100	MEDLINE_EMBASE	The role of the pharmacist in optimizing pharmacotherapy in older people.
#101	MEDLINE_EMBASE	An innovative approach to pharmacy management in a state correctional system.
#102	MEDLINE_EMBASE	Pharmaceutical technology assessment: Perspectives from payers.
#103	MEDLINE_EMBASE	A cost-effectiveness evaluation of hospital discharge counseling by pharmacists.
#104	MEDLINE_EMBASE	Effects of a pharmacy-care program on adherence and outcomes.
#105	MEDLINE_EMBASE	Economic and clinical impact of a pharmacy resident at a large academic medical center.
#106	MEDLINE_EMBASE	"Brown bag" CMRs: Review of the medicine cabinet.
#107	MEDLINE_EMBASE	Abstracts from the Quality of Care and Outcomes Research in Cardiovascular Disease and Stroke 2012 Scientific Sessions, QCOR 2012.
#108	MEDLINE_EMBASE	Evaluation of a pharmacist culture review process in an Emergency Department.
#109	MEDLINE_EMBASE	Systematic review of diabetes disease management interventions.
#110	MEDLINE_EMBASE	White paper on expanding the role of pharmacists in chronic obstructive pulmonary disease.
#111	MEDLINE_EMBASE	Pharmacist-provided immunization compensation and recognition: White paper summarizing APhA/AMCP stakeholder meeting.
#112	MEDLINE_EMBASE	Inventory management in pharmacy practice: A review of literature.
#113	MEDLINE_EMBASE	Factors affecting the cost effectiveness of antibiotics.
#114	MEDLINE_EMBASE	ASHP guidelines on emergency medicine pharmacist services.
#115	MEDLINE_EMBASE	Is there a benefit to having a clinical oncology pharmacist on staff at a community oncology clinic?.
#116	MEDLINE_EMBASE	Impacting readmission rates and patient satisfaction: Results of a discharge pharmacist pilot program.
#117	MEDLINE_EMBASE	Remote order entry services: Providing 24-hour pharmaceutical care for small to medium sized hospitals.
#118	MEDLINE_EMBASE	Pharmacist-managed telephone clinic review of antidementia medication effectiveness.
#119	MEDLINE_EMBASE	Adherence to medication under mandatory and voluntary mail benefit designs.
#120	MEDLINE_EMBASE	Cost-effectiveness of interventions to enhance medication Adherence in Psychiatric Patients: A Systematic Review.
#121	MEDLINE_EMBASE	A cost effectiveness analysis of thiopurine methyltransferase testing for guiding 6-mercaptopurine dosing in children with acute lymphoblastic leukemia.
#122	MEDLINE_EMBASE	The roles of community pharmacists in cardiovascular disease prevention and management.
#123	MEDLINE_EMBASE	Use of pharmacy delivery robots in intensive care units.
#124	MEDLINE_EMBASE	Development and implementation of a pharmacist-managed clinical pharmacogenetics service.
#125	MEDLINE_EMBASE	Role of community pharmacists in asthma - Australian research highlighting pathways for future primary care models.
#126	MEDLINE_EMBASE	Lightening the load? A systematic review of community pharmacy-based weight management interventions.
#127	MEDLINE_EMBASE	Role of collaborative care models including pharmacists in improving blood pressure management in chronic kidney disease patients.
#128	MEDLINE_EMBASE	Pharmaceutical step-therapy interventions: A critical review of the literature.
#129	MEDLINE_EMBASE	Interdisciplinary patient care in the intensive care unit: Focus on the pharmacist.
#130	MEDLINE_EMBASE	Payment for clinical pharmacy services revisited.
#131	MEDLINE_EMBASE	Effectiveness of pharmacy interventions in improving availability of essential medicines at the primary healthcare level.

#132	MEDLINE_EMBASE	Joint Forces Pharmacy Seminar, JFPS 2011 Abstracts.
#133	MEDLINE_EMBASE	Late-Breaking Clinical Trial and Clinical Science: Special Reports Abstracts from the American Heart Association's Scientific Sessions 2011.
#134	MEDLINE_EMBASE	Interventions to improve patients compliance with lipid lowering medications: A systematic review of the literature.
#135	MEDLINE_EMBASE	A retrospective review of medication orders entered for emergency department patients within an academic teaching medical center for quality assurance.
#136	MEDLINE_EMBASE	2011 American Society of Consultant Pharmacists, ASCP Annual Meeting and Exhibition.
#137	MEDLINE_EMBASE	Literature review of randomized, controlled studies of theimpact of pharmacists' interventions to improve patient outcomes.
#138	MEDLINE_EMBASE	Comparative-effectiveness of pharmacist interventions using a decision modeling approach.
#139	MEDLINE_EMBASE	An evaluation of clinical pharmacist contributions in paediatrics.
#140	MEDLINE_EMBASE	A structured literature review of the direct costs of adult systemic lupus erythematosus in the US.
#141	MEDLINE_EMBASE	Blood pressure kiosks for medication therapy management programs: Business opportunity for pharmacists.
#142	MEDLINE_EMBASE	Billing for pharmacists' cognitive services in physicians' offices: Multiple methods of reimbursement.
#143	MEDLINE_EMBASE	Rational use of medicine in the pediatric age group: A summary on the role of clinical pharmacists.
#144	MEDLINE_EMBASE	The Effectiveness of Pharmacist Interventions on Asthma Management: A Systematic Review.
#145	MEDLINE_EMBASE	Systematic review of pharmacist interventions to improve adherence to oral antidiabetic medications in people with type 2 diabetes.
#146	MEDLINE_EMBASE	Committee opinion no 544: Over-the-counter access to oral contraceptives.
#147	MEDLINE_EMBASE	The comparative economic burden of mild, moderate, and severe fibromyalgia: Results from a retrospective chart review and cross-sectional survey of working-age U.S. adults.
#148	MEDLINE_EMBASE	Recent trends on the future of graduate education in the pharmaceutical sciences and research.
#149	MEDLINE_EMBASE	The team education and adherence monitoring (TEAM) trial: Pharmacy interventions to improve hypertension control in blacks.
#150	MEDLINE_EMBASE	A review of remediation programs in pharmacy and other health professions.
#151	MEDLINE_EMBASE	Implementation of a telepharmacy service to provide round-the-clock medication order review by pharmacists.
#152	MEDLINE_EMBASE	Operation of a radiopharmacy for a clinical trial.
#153	MEDLINE_EMBASE	Value of conducting pharmacy residency training - The organizational perspective.
#154	MEDLINE_EMBASE	Symposium on roles of and cooperation between academic- and practice-based pharmacy clinicians.
#155	MEDLINE_EMBASE	Budgetary impact of treatment with adjuvant imatinib for 1 year following surgical resection of kit-positive localized gastrointestinal stromal tumors.
#156	MEDLINE_EMBASE	Formulary review of 2 new biologic agents: Tocilizumab for rheumatoid arthritis and ustekinumab for plaque psoriasis.
#157	MEDLINE_EMBASE	Establishing a central order entry system for delivering telepharmacy services to remote rural hospitals.
#158	MEDLINE_EMBASE	Health care policy and community pharmacy: Implications for the New Zealand primary health care sector.
#159	MEDLINE_EMBASE	Potential cost savings for a health system-based employee outpatient pharmacy.
#160	MEDLINE_EMBASE	Prophylaxis of cervical cancer and related cervical disease: A review of the cost-effectiveness of vaccination against oncogenic HPV types.
#161	MEDLINE_EMBASE	UK interventions to control medicines wastage: A critical review.
#162	MEDLINE_EMBASE	Patient perspective, complexities, and challenges in managed care.
#163	MEDLINE_EMBASE	Empirical view of opioid dependence.
#164	MEDLINE_EMBASE	Introduction: Landscape of opioid dependence.
#165	MEDLINE_EMBASE	Strategies for improving asthma outcomes: A case-based review of successes and pitfalls.
#166	MEDLINE_EMBASE	Effective use of workload and productivity monitoring tools in health-system pharmacy, part 2.
#167	MEDLINE_EMBASE	Evidence-based, multidisciplinary approach to the development of a Crotalidae polyvalent antivenin (CroFab) protocol at a university hospital.
#168	MEDLINE_EMBASE	Current challenges in European oncology pharmacy practice.
#169	MEDLINE_EMBASE	Effective use of workload and productivity monitoring tools in health-system pharmacy, part 1.
#170	MEDLINE_EMBASE	Systematic review of the implementation and evaluation of Pharmaceutical Care in hospitalised patients(Pharmaceutical Care implementation in hospitalised patients. Systematic review). [Spanish]
#171	MEDLINE_EMBASE	Interventions to improve compliance with lipid lowering medications: A systematic review of the literature.
#172	MEDLINE_EMBASE	Evidence used during pharmaceutical technology assessment.
#173	MEDLINE_EMBASE	Willingness to pay for pharmacy services: A review.
#174	MEDLINE_EMBASE	The methodological quality and effectiveness of adherence interventions: A review of diabetes type II interventions.
#175	MEDLINE_EMBASE	The impact of pharmacist intervention on patients' adherence to antidepressant medication and patient-reported outcomes.
#176	MEDLINE_EMBASE	A systematic review to identify specific pharmacists' interventions and outcomes associated with drug therapy.

#177	MEDLINE_EMBASE	A systematic literature review of the direct costs of systemic lupus erythematosus (SLE) in the United States (US).
#178	MEDLINE_EMBASE	Retrospective chart review of a clinical pharmacist's interventions during rounds in a hospice inpatient unit.
#179	MEDLINE_EMBASE	Community pharmacy EHC services: A review of uptake and users in one primary care organisation 2028-2009.
#180	MEDLINE_EMBASE	The role of pharmacotherapy and managed care pharmacy interventions in the treatment of ADHD.
#182	MEDLINE_EMBASE	Current and future directions in MS management: Key considerations for managed care pharmacists.
#183	MEDLINE_EMBASE	Evaluation and justification of clinical pharmacy services.
#184	MEDLINE_EMBASE	Review of prasugrel for the secondary prevention of atherothrombosis.
#185	MEDLINE_EMBASE	Cognitive services in community pharmacy: Pharmaceutical interventions and medication review. [Slovene]
#186	MEDLINE_EMBASE	Critical review of prasugrel for formulary decision makers.
#187	MEDLINE_EMBASE	Implementing a comprehensive, 24-hour emergency department pharmacy program.
#188	MEDLINE_EMBASE	Establishing the role of the pharmacist in the patient-centered medical home... The opportunity is now.
#189	MEDLINE_EMBASE	Economic burden of prescription opioid misuse and abuse.
#190	MEDLINE_EMBASE	Improving cost-effectiveness of and outcomes from drug therapy in patients with atrial fibrillation in managed care: Role of the pharmacist.
#191	MEDLINE_EMBASE	Pharmacologic management of atrial fibrillation: Established and emerging options.
#192	MEDLINE_EMBASE	Clinical, economic, and quality of life impact of atrial fibrillation.
#193	MEDLINE_EMBASE	AMCP guide to pharmaceutical payment methods, 2009 update.
#194	MEDLINE_EMBASE	Provision of clinical pharmacy services in two safety net provider settings.
#195	MEDLINE_EMBASE	Health technology assessment (HTA) of medical devices in the Veneto region, Italy. [Italian]
#196	MEDLINE_EMBASE	ERISA preemption and the question of pharmacy benefit managers' fiduciary duty.
#197	MEDLINE_EMBASE	Effect of clinical pharmacists on care in the emergency department: A systematic review.
#198	MEDLINE_EMBASE	The feasibility of providing community pharmacy-based services for alcohol misuse: A literature review.
#199	MEDLINE_EMBASE	Collaborative strategies for improving clinical resource utilization: How pharmacists can make a difference.
#200	MEDLINE_EMBASE	APOSTAT - an electronic database for documentation and analysis of pharmaceutical interventions. [German]
#201	MEDLINE_EMBASE	A blueprint for pharmacy benefit managers to increase value.
#202	MEDLINE_EMBASE	Review of the clinical debate regarding interventions for multiple sclerosis.
#203	MEDLINE_EMBASE	Budgetary impact of varenicline in smoking cessation in the United Kingdom.
#204	MEDLINE_EMBASE	Pharmacist involvement in improving asthma outcomes in various healthcare settings: 1997 to present.
#205	MEDLINE_EMBASE	Medication review and patient counselling at discharge from the hospital by community pharmacists.
#206	MEDLINE_EMBASE	CSHP Summer Educational Sessions (SES) 2009: Poster Abstracts.
#207	MEDLINE_EMBASE	A retrospective review of erythrocyte stimulating agents (ESA) usage in pharmacy claims data.
#208	MEDLINE_EMBASE	Intersectoral collaboration to enable bridging education for pharmacists: The international pharmacy graduate program in Ontario, Canada.
#209	MEDLINE_EMBASE	A 10-year-review (1998 - 2007) on 3449 cases of osteoporotic hip fractures: trend of hospitalization and inpatient costs. [Chinese]
#210	MEDLINE_EMBASE	Testing the long term stability of vancomycin ophthalmic solution.
#211	MEDLINE_EMBASE	Pharmacy education in the context of Australian practice.
#212	MEDLINE_EMBASE	Optimal treatment of chronic constipation in managed care: Review and roundtable discussion.
#213	MEDLINE_EMBASE	A primer on critical care pharmacy services.
#214	MEDLINE_EMBASE	Targeting low HDL-cholesterol to decrease residual cardiovascular risk in the managed care setting.
#215	MEDLINE_EMBASE	Economic and clinical impact of multiple myeloma to managed care.
#216	MEDLINE_EMBASE	Rebates and spreads: Pharmacy benefit management practices and corporate citizenship.
#217	MEDLINE_EMBASE	Special needs plans.
#218	MEDLINE_EMBASE	Pay-for-performance initiatives.
#219	MEDLINE_EMBASE	Medication therapy management.
#220	MEDLINE_EMBASE	Overview of current medicare part D offerings.
#221	MEDLINE_EMBASE	Medication compliance, adherence, and persistence: Current status of behavioral and educational interventions to improve outcomes.
#222	MEDLINE_EMBASE	Clinical pharmacy services in the home.
#223	MEDLINE_EMBASE	Therapy analysis and evidenced-based treatment guidelines (including review of antiviral medications).
#224	MEDLINE_EMBASE	Managing type 2 diabetes: Going beyond glycemic control.
#225	MEDLINE_EMBASE	Pharmaceutical care in community pharmacies: Practice and research in Estonia.

#226	MEDLINE_EMBASE	The state of health economic and pharmacoeconomic evaluation research in Zimbabwe: A review.
#227	MEDLINE_EMBASE	Managed care perspective on three new agents for type 2 diabetes.
#228	MEDLINE_EMBASE	Benefit design innovation to manage speciatly pharmaceuticals.
#229	MEDLINE_EMBASE	Managing the intersection of medical and pharmacy benefits.
#230	MEDLINE_EMBASE	The promise of specialty pharmaceuticals: Are they worth the price?
#231	MEDLINE_EMBASE	Economic effects of clinical pharmacy interventions: A literature review.
#232	MEDLINE_EMBASE	Pharmacist-led interventions for the management of cardiovascular disease: Opportunities and obstacles.
#233	MEDLINE_EMBASE	A systematic review of remuneration systems for clinical pharmacy care services.
#234	MEDLINE_EMBASE	Effects of pharmacist outpatient interventions on adults with diabetes mellitus: A systematic review.
#235	MEDLINE_EMBASE	Economic burden in direct costs of concomitant chronic obstructive pulmonary disease and asthma in a Medicare Advantage population.
#236	MEDLINE_EMBASE	Operationalizing MTM through the use of Health Information Technology.
#237	MEDLINE_EMBASE	Information technology for the treatment of diabetes: Improving outcomes and controlling cost.
#238	MEDLINE_EMBASE	Medicare Part D: Selected issues for plan sponsors, pharmacists, and beneficiaries in 2008.
#239	MEDLINE_EMBASE	Dual degree programs at the University of Kentucky College of Pharmacy.
#240	MEDLINE_EMBASE	Achievement of USP chapter <797> compliance by a 35-bed rural hospital: A case study.
#241	MEDLINE_EMBASE	Implications of pharmacogenomics in the current and future treatment of asthma.
#242	MEDLINE_EMBASE	Management of chronic diseases in the 21st century: The emerging role of specialty pharmacies.
#243	MEDLINE_EMBASE	New strategies in controlling drug resistance.
#244	MEDLINE_EMBASE	Targeted chronic myeloid leukemia therapy: Seeking a cure.
#245	MEDLINE_EMBASE	Chronic myeloid leukemia in 2007.
#246	MEDLINE_EMBASE	Pharmacy input in medications review improves prescribing and cost-efficiency in care homes.
#247	MEDLINE_EMBASE	Medicare challenges and solutions - Reimbursement issues in treating the patient with colorectal cancer.
#248	MEDLINE_EMBASE	Epidemiology, disease progression, and economic burden of colorectal cancer.
#249	MEDLINE_EMBASE	Asthma management guidelines: Updates, advances, and new options.
#250	MEDLINE_EMBASE	What employers need to know about the changing landscape of the pharmaceutical marketplace.
#251	MEDLINE_EMBASE	Treatment-resistant depression: Managed care considerations.
#252	MEDLINE_EMBASE	Outcomes of secondary hyperparathyroidism in chronic kidney disease and the direct costs of treatment.
#253	MEDLINE_EMBASE	Medication errors in older adults: A review of recent publications.
#254	MEDLINE_EMBASE	Adverse drug reactions and current state of drug regimen review in nursing facilities: Need for a change?.
#255	MEDLINE_EMBASE	Pharmacists' role in the post-discharge management of patients with heart failure: A literature review.
#256	MEDLINE_EMBASE	Pharmacoeconomic analysis of clopidogrel in secondary prevention of coronary artery disease.
#257	MEDLINE_EMBASE	Providing patient care in community pharmacies: Practice and research in Finland.
#258	MEDLINE_EMBASE	30 Ways to control health care costs.
#259	MEDLINE_EMBASE	Medicare Part D: Selected issues for pharmacists and beneficiaries in 2007.
#260	MEDLINE_EMBASE	Use of low-molecular-weight heparin during dental extractions in a medicaid population.
#261	MEDLINE_EMBASE	Drug-related problems in hospitals: A review of the recent literature.
#262	MEDLINE_EMBASE	Pharmaceutical care in community pharmacies: Practice and research in Denmark.
#263	MEDLINE_EMBASE	Clinical pharmacy services in a home-based palliative care program.
#264	MEDLINE_EMBASE	Total knee replacement delayed with hylan G-F 20 use in patients with grade IV osteoarthritis.
#265	MEDLINE_EMBASE	Type 2 diabetes and cardiovascular disease: Reducing the risk.
#266	MEDLINE_EMBASE	A randomised controlled trial of the effects of note-based medication review by community pharmacists on prescribing of cardiovascular drugs in general practice.
#267	MEDLINE_EMBASE	Evaluating the costs of brand-name and generic prescriptions dispensed by mail versus retail.
#268	MEDLINE_EMBASE	Drug utilization review in an extended care facility. 1974.
#269	MEDLINE_EMBASE	Continuing education: Geriatric care: From adverse events to urinary incontinence.
#270	MEDLINE_EMBASE	Economic impact of antidiabetic medications and glycemic control on managed care organizations: A review of the literature.
#271	MEDLINE_EMBASE	Medication adherence: A challenge for patients with postmenopausal osteoporosis and other chronic illnesses.
#272	MEDLINE_EMBASE	Prescription drug therapies for prevention and treatment of postmenopausal osteoporosis.
#273	MEDLINE_EMBASE	Introduction: Postmenopausal osteoporosis as a major public health issue.
#274	MEDLINE_EMBASE	Review of eight pharmacoeconomic studies of the value of biologic DMARDs (adalimumab, etanercept, and infliximab) in the management of rheumatoid arthritis.

#275	MEDLINE_EMBASE	Prevalence of drug-related problems and cost-savings opportunities in Medicaid high utilizers identified by a pharmacist-run drug regimen review center.
#276	MEDLINE_EMBASE	A comparison of clinical practice guidelines in the initial pharmacological management of new-onset epilepsy in adults.
#277	MEDLINE_EMBASE	Specialty pharmacy cost management strategies of private health care payers.
#278	MEDLINE_EMBASE	What are incretins, and how will they influence the management of type 2 diabetes?
#279	MEDLINE_EMBASE	Economic considerations in managing patients with chronic stable angina.
#280	MEDLINE_EMBASE	From asthma severity to asthma control: Identification of new guidelines.
#281	MEDLINE_EMBASE	The effects of pharmacist interventions on patients with polypharmacy. [Spanish]
#282	MEDLINE_EMBASE	Effect of attitudes and perceptions of independent community pharmacy owners/managers on the comprehensiveness of strategic planning.
#283	MEDLINE_EMBASE	Community pharmacy services in Portugal.
#284	MEDLINE_EMBASE	Current state of community pharmacy in Japan: Practice, research, and future opportunities or challenges.
#285	MEDLINE_EMBASE	New recommandations from the Institute of Medicine on preventing medication errors.
#286	MEDLINE_EMBASE	Pharmaceutical care in community pharmacies: Practice and research in the US.
#287	MEDLINE_EMBASE	Pharmacy-managed anticoagulation: Assessment of in-hospital efficacy and evaluation of financial impact and community acceptance.
#288	MEDLINE_EMBASE	Bringing the benefits of anticoagulation management services to the community.
#289	MEDLINE_EMBASE	Potential role of the community liaison pharmacist: Stakeholder views.
#290	MEDLINE_EMBASE	Substance abuse and psychiatric disorders in HIV-positive patients: Epidemiology and impact on antiretroviral therapy.
#291	MEDLINE_EMBASE	Evaluating outpatient pharmacy services: A literature review of specialist heart failure services.
#292	MEDLINE_EMBASE	Personal digital assistant (PDA) clinical intervention documentation system: Development implementation, and comparison to a previous paper-based system.
#293	MEDLINE_EMBASE	Projecting future drug, expenditures - 2006.
#294	MEDLINE_EMBASE	Effective communication of antimicrobial susceptibility data by pathologists to clinicians.
#295	MEDLINE_EMBASE	Clinical monograph for drug formulary review: erectile dysfunction agents.
#296	MEDLINE_EMBASE	Medication therapy management services: a critical review.
#297	MEDLINE_EMBASE	Essential steps and practical applications for database studies.
#298	MEDLINE_EMBASE	Clinical monograph for drug formulary review: systemic agents for psoriasis/psoriatic arthritis.
#299	MEDLINE_EMBASE	The evolving use of cost-effectiveness analysis in formulary management within the department of veterans affairs.
#300	MEDLINE_EMBASE	Pharmacy utilization and the Medicare Modernization Act.
#302	MEDLINE_EMBASE	Community pharmacy services to optimise the use of medications for mental illness: A systematic review.
#303	MEDLINE_EMBASE	Graduate studies as part of a career path for Saskatchewan pharmacy students.
#304	MEDLINE_EMBASE	Effectiveness and acceptability of community pharmacy-based interventions in type 2 diabetes: A critical review of intervention design, pharmacist and patient perspectives.
#305	MEDLINE_EMBASE	Maximizing your profits with pharmacy control strategies.
#306	MEDLINE_EMBASE	Deregulating the pharmacy market: The case of Iceland and Norway.
#307	MEDLINE_EMBASE	Understanding the impacts of the Medicare Modernization Act: Concerns of congressional staff.
#308	MEDLINE_EMBASE	The roles of community pharmacists in managing patients with diabetes: Perceptions of health care professionals in Nova Scotia.
#309	MEDLINE_EMBASE	The growth of psychopharmacology in the 1990s: Evidence-based practice or irrational exuberance.
#310	MEDLINE_EMBASE	Devices for ambulatory and home monitoring of blood pressure, lipids, coagulation, and weight management, part 2.
#311	MEDLINE_EMBASE	The medicare prescription drug benefit and managed care pharmacy.
#312	MEDLINE_EMBASE	Devices for ambulatory and home monitoring of blood pressure, lipids, coagulation, and weight management, part 1.
#313	MEDLINE_EMBASE	Patient education and treatment strategies implemented at a pharmacist-managed hepatitis C virus clinic.
#314	MEDLINE_EMBASE	The involvement of pharmacists in cardiac rehabilitation: A review of the literature.
#315	MEDLINE_EMBASE	Rational use of medicines - An important issue in pharmaceutical policy.
#316	MEDLINE_EMBASE	The clinical effectiveness initiative: A pharmacy analysis to improve outcomes and costs of drug therapy.
#317	MEDLINE_EMBASE	Is oncology compatible with specialty pharmacy?
#318	MEDLINE_EMBASE	Alternative strategies for medicare payment of outpatient prescription drugs - Part B and beyond.
#319	MEDLINE_EMBASE	Managed care pharmacy and medication safety: The fourth estate.
#320	MEDLINE_EMBASE	The WellPoint outcomes based Formulary^{SM}: Enhancing the health technology assessment process.
#321	MEDLINE_EMBASE	Pharmacy Benefits Management in the Veterans Health Administration: 1995 to 2003.

#322	MEDLINE_EMBASE	A pharmacoeconomic model for making value-based decisions about serotonin reuptake inhibitors.
#323	MEDLINE_EMBASE	Approaches to pharmacy benefit management and the impact of consumer cost sharing.
#324	MEDLINE_EMBASE	Quality improvement in the continuum of care: impact of atherothrombosis in managed care pharmacy.
#325	MEDLINE_EMBASE	Current status of prospective drug utilization review.
#326	MEDLINE_EMBASE	A literature review of cardiovascular disease management programs in managed care populations.
#327	MEDLINE_EMBASE	Advancements in the treatment of psoriasis: role of biologic agents.
#328	MEDLINE_EMBASE	Allergic rhinitis, asthma, and rhinosinusitis: diseases of the integrated airway.
#329	MEDLINE_EMBASE	The burden of illness of irritable bowel syndrome: current challenges and hope for the future.
#330	MEDLINE_EMBASE	Evidence-based and value-based formulary guidelines.
#331	MEDLINE_EMBASE	Framework for pharmacy services quality improvement--a bridge to cross the quality chasm. Part I. The opportunity and the tool.
#332	MEDLINE_EMBASE	Pharmacy residency training in academic medical centers.
#333	MEDLINE_EMBASE	Reflexions on the organisation of the purchase of single use supplies associated to biomedical equipments: Respective roles of the biomedical service and pharmacy. [French]
#334	MEDLINE_EMBASE	Pharmacist prescribing: Review of the literature.
#335	MEDLINE_EMBASE	Quantitative evaluation of hospital drug formularies in hospitals in Slovak Republic. [Slovak]
#336	MEDLINE_EMBASE	Blood, sweat, and tears: Wasted by medicare's missed opportunity.
#337	MEDLINE_EMBASE	A literature review to identify interventions to improve the use of medicines in children.
#338	MEDLINE_EMBASE	Naltrexone and pharmacy benefit management.
#339	MEDLINE_EMBASE	Evaluation of a community pharmacy reimbursement model for cognitive services-experiment and survey.
#340	MEDLINE_EMBASE	A comprehensive system of pharmaceutical care for drug misusers.
#341	MEDLINE_EMBASE	Optimizing drug prescribing in managed care populations: Improving clinical and economic outcomes.
#342	MEDLINE_EMBASE	Assessment of a retrospective drug utilization review alert letter program.
#343	MEDLINE_EMBASE	Specialty pharmaceuticals: Delivering comprehensive integrated care.
#344	MEDLINE_EMBASE	Can clinical pharmacy services have a positive impact on drug-related problems and health outcomes in community-based older adults?.
#345	MEDLINE_EMBASE	Prescription Drug Benefits: Cost Management Issues for Medicare.
#346	MEDLINE_EMBASE	Clinical and Economic Impact of Pharmacy Services in a Low-Income Family Practice Clinic Within a Community Hospital Setting.
#347	MEDLINE_EMBASE	Evidence-Based Core Clinical Pharmacy Services in United States Hospitals in 2020: Services and Staffing.
#348	MEDLINE_EMBASE	Fleetwood Project Phase III: Developing a Payment Methodology.
#349	MEDLINE_EMBASE	A Pharmacist-Physician Antibiotic Support Team.
#350	MEDLINE_EMBASE	Implementing pharmaceutical care in an inner city pharmacy: Hypertension management and elderly African Americans.
#351	MEDLINE_EMBASE	Considerations in Darbepoetin alfa Cost and Reimbursement: A Model for Pharmacy Managers.
#352	MEDLINE_EMBASE	Pharmacist-managed diabetes service in a family medicine practice improves patient outcomes.
#353	MEDLINE_EMBASE	A Review of Clinical Pharmacy Interventions Prior to Implementation of a Personal Digital Assistant Intervention Program in a Community Hospital.
#354	MEDLINE_EMBASE	Impact of Disease Management on Cost-Effectiveness of Medicare Spending.
#355	MEDLINE_EMBASE	Weight variability of scored and unscored split psychotropic drug tablets.
#356	MEDLINE_EMBASE	Outreach pharmacy service in old age homes: A Hong Kong experience.
#357	MEDLINE_EMBASE	The Academy of Managed Care Pharmacy Format for Formulary Submissions: An evolving standard - A Foundation for Managed Care Pharmacy task force report.
#358	MEDLINE_EMBASE	Reduction of polypharmacy in the elderly: A systematic review of the role of the pharmacist.
#359	MEDLINE_EMBASE	Pharmacy data in the VA health care system.
#360	MEDLINE_EMBASE	A pharmacy-managed intravenous to enteral proton-pump inhibitor conversion program.
#361	MEDLINE_EMBASE	Use of pharmacoeconomics information - Report of the ISPOR Task Force on use of pharmacoeconomic/health economic information in health-care decision making.
#362	MEDLINE_EMBASE	Public health interventionists, penny capitalists, or sources of risk?: Assessing street syringe sellers in Hartford, Connecticut.
#363	MEDLINE_EMBASE	Quality use of medicines in aged-care facilities in Australia.
#364	MEDLINE_EMBASE	Systematic review of the effectiveness of community pharmacy-based interventions to reduce risk behaviours and risk factors for coronary heart disease.
#365	MEDLINE_EMBASE	The impact of critical care pharmacists on enhancing patient outcomes.
#366	MEDLINE_EMBASE	Designing and implementing an effective specialty pharmacy service program.
#367	MEDLINE_EMBASE	An overview of community pharmacist interventions: Assessing cost-effectiveness and patients' willingness to pay.
#368	MEDLINE_EMBASE	Creating a hospice pharmacy and therapeutics committee.

#369	MEDLINE_EMBASE	Evaluation of the compliance with law duties in prescriptions of a hospital pharmacy service from Ouro Preto and implications in the patients assistance quality. [Portuguese]
#370	MEDLINE_EMBASE	Experiences with pharmacy benefit management programmes in the USA.
#371	MEDLINE_EMBASE	The critical care pharmacist: An essential intensive care practitioner.
#372	MEDLINE_EMBASE	PBMs: Ripe for regulation.
#373	MEDLINE_EMBASE	The academy of managed care pharmacy's guidance on the use of clinical and economic evaluation data to support drug formulary decisions.
#374	MEDLINE_EMBASE	Etanercept and its implications for managed care.
#375	MEDLINE_EMBASE	Building the outcomes-based formulary.
#376	MEDLINE_EMBASE	A cost analysis of switching calcium channel blockers: A one-year post-formulary decision review.
#377	MEDLINE_EMBASE	Pharmacy benefit managers role in facilitating Medicare beneficiary access to pharmaceutical care.
#378	MEDLINE_EMBASE	American Consumers Give Mail Service Pharmacy a Strong Vote of Confidence.
#379	MEDLINE_EMBASE	Managed care pharmacist: Transforming health care through regulatory action (not a managed care story).
#380	MEDLINE_EMBASE	Making sense of outpatient pharmacy management: How to control pharmacy costs while delivering quality care.
#381	MEDLINE_EMBASE	Pharmacy benefit managers and medicare beneficiary access to prescription drugs.
#382	MEDLINE_EMBASE	Intensive care unit antimicrobial resistance and the role of the pharmacist.
#383	MEDLINE_EMBASE	Cost avoidance and clinical benefits derived from a pharmacy-managed anemia program.
#384	MEDLINE_EMBASE	Studies demonstrating improved outcomes in patients with asthma: A 10-year review.
#385	MEDLINE_EMBASE	Expanding the roles of outpatient pharmacists: effects on health services utilisation, costs, and patient outcomes.
#386	MEDLINE_EMBASE	The impact of managed care on pharmacy practice.
#387	MEDLINE_EMBASE	The Medicare prescription drug benefit: how will the game be played?.
#389	MEDLINE_EMBASE	The timmins project: A study in managed care.
#390	MEDLINE_EMBASE	Improving pharmacy supply-chain management in the operating room.
#391	MEDLINE_EMBASE	As the debate over a Medicare drug benefit intensifies, the role of pharmacy benefit managers is in the spotlight.
#392	MEDLINE_EMBASE	Cost-benefit analysis of a clinical pharmacist's presence on a post-liver transplant service (confessions of some accidental hermeneuticians).
#393	MEDLINE_EMBASE	Inadequate reimbursement for pharmacist cognitive services.
#394	MEDLINE_EMBASE	Impact of pharmacists providing a prescription review and monitoring service in ambulatory care or community practice.
#395	MEDLINE_EMBASE	Cost analysis of pharmacy-based aminoglycoside regimens: Once-daily vs. multiple daily dosing.
#396	MEDLINE_EMBASE	Prescription drug use and seniors.
#397	MEDLINE_EMBASE	Improved phenytoin monitoring through pharmacist intervention.
#398	MEDLINE_EMBASE	Economic and humanistic impact of adverse events associated with CSFs.
#399	MEDLINE_EMBASE	Drug of compassionate use: More responsibility for the hospital pharmacy services. [Spanish]
#400	MEDLINE_EMBASE	Practical hospital pharmacy management: Drug distribution.
#401	MEDLINE_EMBASE	Practical hospital pharmacy management: Management strategies and methods.
#402	MEDLINE_EMBASE	Uses and abuses of prescription drug information in pharmacy benefits management programs.
#403	MEDLINE_EMBASE	Use of contingent valuation to place a monetary value on pharmacy services: An overview and review of the literature.
#404	MEDLINE_EMBASE	Dollars and sense: a practical guide to cost analysis for hospital epidemiology and infection control.
#405	MEDLINE_EMBASE	Pharmaceutical care intervention documentation program and related cost savings at a University Hospital.
#406	MEDLINE_EMBASE	Managed care pharmacist: Trends in managed care pharmacy benefits.
#407	MEDLINE_EMBASE	Managed care pharmacist: National health care expenditures remain constant.
#408	MEDLINE_EMBASE	Kickbacks, courtesies or cost-effectiveness?: Application of the medicare antikickback law to the marketing and promotional practices of drug and medical device manufacturers.
#409	MEDLINE_EMBASE	Economic evaluation of pharmacy services - Fact or fiction?
#410	MEDLINE_EMBASE	Pharmacy benefit management companies: Dimensions of performance.
#411	MEDLINE_EMBASE	Interventions in antimicrobial therapy made by infectious diseases clinical pharmacy specialists.
#412	MEDLINE_EMBASE	Out patient management of venous thromboembolism.
#413	MEDLINE_EMBASE	Ensuring a role in diabetes disease management programs.
#414	MEDLINE_EMBASE	The potential for self-interested behavior by pharmaceutical manufacturers through vertical integration with pharmacy benefit managers: The need for a new regulatory approach.
#415	MEDLINE_EMBASE	Impact of trauma stress ulcer prophylaxis guidelines on drug cost and frequency of major gastrointestinal bleeding.
#416	MEDLINE_EMBASE	The effect of managed care on prescription drug costs and benefits.
#417	MEDLINE_EMBASE	Data-based pharmacy utilization review identifies those at-risk, improves care, cuts utilization.

#418	MEDLINE_EMBASE	Experience with external review panels to validate a large clinical pharmacy intervention study.
#419	MEDLINE_EMBASE	Pharmacist-managed anticoagulation clinics: A review.
#420	MEDLINE_EMBASE	A review of the pharmacoeconomics of pharmaceutical care.
#421	MEDLINE_EMBASE	Patient consultation in a managed care setting: Guiding pharmacy into the future.
#422	MEDLINE_EMBASE	Pharmaceutical care evaluated: The value of your services.
#423	MEDLINE_EMBASE	The effect of pharmacy benefit manager clinical programs and services on access to prescription drug benefit coverage.
#424	MEDLINE_EMBASE	Introduction to biothics (8): The right to pharmacy service. [Spanish]
#425	MEDLINE_EMBASE	The vertically integrated pharmacy department.
#426	MEDLINE_EMBASE	Cost-effectiveness of clinical pharmacist intervention: An analysis of conscious sedation in the ICU.
#427	MEDLINE_EMBASE	A collaborative approach to cost-containment in the surgical suite.
#428	MEDLINE_EMBASE	Integrating asthma management into pharmacy practice.
#429	MEDLINE_EMBASE	Overview of the hospital formulary systems in Hong Kong: Princess Margaret Hospital as a baseline.
#430	MEDLINE_EMBASE	Financial risk management of pharmacy benefits.
#431	MEDLINE_EMBASE	Remaking the rules: Pharmacy reengineering opportunities and challenges in a small community hospital.
#432	MEDLINE_EMBASE	Drug data debate.
#433	MEDLINE_EMBASE	Nephrology pharmacy practice in the outpatient dialysis setting.
#434	MEDLINE_EMBASE	Managed care pharmacist. Pharmacotherapeutic algorithms: A new opportunity for pharmacists.
#435	MEDLINE_EMBASE	Pharmaceutical services in the Department of Veterans Affairs.
#436	MEDLINE_EMBASE	Providing pharmaceutical care in an ambulatory care pharmacy within an academic medical center.
#437	MEDLINE_EMBASE	The Iowa On-Line Prospective Drug Utilization Review (OPDUR) demonstration project: Study design, system design, and conceptual models.
#438	MEDLINE_EMBASE	Lipid management: An opportunity for pharmacy service.
#439	MEDLINE_EMBASE	The role of pharmacoeconomics in disease management. A pharmaceutical benefit management company perspective.
#440	MEDLINE_EMBASE	Pharmacist-managed primary care dermatology clinic.
#441	MEDLINE_EMBASE	Decreasing liability potential in the hospital pharmacy.
#442	MEDLINE_EMBASE	The Clinical Pharmacy Services Study. A study of clinical services provided by pharmacists in Ontario Hospitals.
#443	MEDLINE_EMBASE	Projecting future drug expenditures - 1996.
#444	MEDLINE_EMBASE	Effectiveness of Pharmacy-Based Needle/Syringe Exchange Program for People Who Inject Drugs: A Systematic Review and Meta-analysis.
#445	MEDLINE_EMBASE	Cholesterol Point-of-Care Testing for Community Pharmacies: A Review of the Current Literature.
#446	MEDLINE_EMBASE	What is known about community pharmacy supply of naloxone? A scoping review.
#448	MEDLINE_EMBASE	Pharmacist Interventions in the Management of Type 2 Diabetes Mellitus: A Systematic Review of Randomized Controlled Trials.
#450	MEDLINE_EMBASE	Institutional framework for integrated Pharmaceutical Benefits Management: results from a systematic review.
#451	MEDLINE_EMBASE	Clinical pharmacy faculty interventions in a pediatric intensive care unit: an eight-month review.
#452	MEDLINE_EMBASE	Pharmacy interventions on prescribing in nursing homes: from evidence to practice.
#453	MEDLINE_EMBASE	A review of countries' pharmacist-patient communication legal requirements on prescription medications and alignment with practice: Comparison of Nordic countries.
#454	MEDLINE_EMBASE	Randomised evaluation of the Italian medicines use review provided by community pharmacists using asthma as a model (RE I-MUR).
#455	MEDLINE_EMBASE	Role of the Pharmacist in Medication Therapy Management Services in Patients With Osteoporosis.
#457	MEDLINE_EMBASE	Defining and advancing ambulatory care pharmacy practice: it is time to lengthen our stride.
#458	MEDLINE_EMBASE	[Cooperation between general practitioners and pharmacists: a systematic review]. [French]
#459	MEDLINE_EMBASE	An innovative off-campus infusion suite designed to improve experiences of patients with cancer.
#460	MEDLINE_EMBASE	[Assessment, frequency, causes, and prevention of medication errors - a critical analysis]. [German]
#461	MEDLINE_EMBASE	A patient-centered pharmacy services model of HIV patient care in community pharmacy settings: a theoretical and empirical framework.
#462	MEDLINE_EMBASE	Pharmacy benefit managers: evaluating clinical utility in the real world.
#463	MEDLINE_EMBASE	The modeled lifetime cost-effectiveness of published adherence-improving interventions for antihypertensive and lipid-lowering medications.
#464	MEDLINE_EMBASE	Managed care interventions for improving outcomes in acute heart failure syndromes.
#465	MEDLINE_EMBASE	Iatrogenic disease management: moderating medication errors and risks in a pharmacy benefit management environment.
#466	MEDLINE_EMBASE	Pharmacist's role in managing anemia in patients with chronic kidney disease: potential clinical and economic benefits.
#467	MEDLINE_EMBASE	Current trends in pharmacy benefit designs: a threat to disease management in chronic complex diseases.

#468	MEDLINE_EMBASE	Pharmaceutical care in community pharmacies: practice and research in Sweden.
#470	MEDLINE_EMBASE	Cost of a pharmacist-directed intervention to increase treatment of hypercholesterolemia.
#471	MEDLINE_EMBASE	Advances in postoperative pain management: the pharmacy perspective.
#472	MEDLINE_EMBASE	Pharmacist compensation for cognitive services: focus on the physician office and community pharmacy.
#473	MEDLINE_EMBASE	Role of the pharmacist in establishing lipid intervention programs.
#475	MEDLINE_EMBASE	Establishing value in managed care: cost-effectiveness or budgetary impact?
#476	MEDLINE_EMBASE	The managed care pharmacy perspective.
#477	MEDLINE_EMBASE	Combined evidence-based literature analysis and consensus guidelines for stocking of emergency antidotes in the United States.
#478	MEDLINE_EMBASE	The impact of pharmaceutical services in community and ambulatory care settings: evidence and recommendations for future research.
#479	MEDLINE_EMBASE	Opportunities for depression disease management: a pharmacy benefit manager's experience.
#480	MEDLINE_EMBASE	Programs promoting timely sequential antimicrobial therapy: an American perspective.
#481	MEDLINE_EMBASE	Impact of managed care in pharmacy.
#482	MEDLINE_EMBASE	Pharmacoeconomics and therapeutic drug monitoring.
#485	MEDLINE_EMBASE	Stress echocardiography.
#486	MEDLINE_EMBASE	Pharmacists as agents of change for rational drug therapy.
#487	MEDLINE_EMBASE	Pharmacist-managed medication review in a managed care system.
#488	MEDLINE_EMBASE	Projecting future drug expenditures--1994.
#489	MEDLINE_EMBASE	Use of therapeutic drug monitoring in pharmacoeconomics.
#490	MEDLINE_EMBASE	1993 Bibliography: a 32-year literature review on the value and acceptance of ambulatory care provided by pharmacists.
#491	MEDLINE_EMBASE	Assessing the quality of pharmaceutical care. II. Application of concepts of quality assessment from medical care.
#492	MEDLINE_EMBASE	Impact of pharmacist interventions on medication management by the elderly: a review of the literature.
#493	MEDLINE_EMBASE	Ambulatory care pharmacy services: the incomplete agenda.
#494	MEDLINE_EMBASE	Postmarketing surveillance of adverse drug reactions: patient self-monitoring.
#495	MEDLINE_EMBASE	Cefoxitin use review.
#496	MEDLINE_EMBASE	Choices in prescription-drug benefit programs: mail versus community pharmacy services.
#497	MEDLINE_EMBASE	Acceptance of pharmacists' suggestions by prescribers: a literature review.
#498	MEDLINE_EMBASE	Ciprofloxacin drug utilization review and prospective drug use evaluation.
#499	MEDLINE_EMBASE	Management consultant review of three departments of pharmacy to identify cost-reduction opportunities.
#500	MEDLINE_EMBASE	Streamlining antimicrobial therapy through pharmacists' review of order sheets.
#501	MEDLINE_EMBASE	Economic assessment of peptic ulcer disease treatments.
#502	MEDLINE_EMBASE	A review of cost-benefit and cost-effectiveness analyses of clinical pharmacy services.
#503	MEDLINE_EMBASE	Unresolved issues in the future of pharmacy.
#504	MEDLINE_EMBASE	Monetary valuation of benefits of pharmacy services.
#505	MEDLINE_EMBASE	An eleven-year review of the pharmacy literature: documentation of the value and acceptance of clinical pharmacy.
#506	MEDLINE_EMBASE	Total formulary review--the easy way.
#507	MEDLINE_EMBASE	Controlling financial variables--changing prescribing patterns.
#508	MEDLINE_EMBASE	Reimbursement for nondistributive pharmaceutical services in hospitals.
#509	MEDLINE_EMBASE	A systematic review to assess adherence and persistence with statins.
#510	MEDLINE_EMBASE	Use of telemedicine to enhance pharmacist services in the nursing facility.
#511	MEDLINE_EMBASE	Roles and impacts of pharmacist in oncology: A review of literature. [French]
#513	MEDLINE_EMBASE	Interventions to improve adherence to lipid-lowering medication.
#514	MEDLINE_EMBASE	American College of Clinical Pharmacy 2016 Annual Meeting, ACCP 2016.
#515	MEDLINE_EMBASE	Clinical pharmacist impact in outpatient oncology practices: A systematic review.
#516	MEDLINE_EMBASE	Deprescribing of medicines in care homes - Primary care pharmacists' practices and perspectives.
#517	MEDLINE_EMBASE	Risk factors for developing drug-related problems in patients with cardiovascular diseases attending Gondar University Hospital, Ethiopia.
#518	MEDLINE_EMBASE	Impact of pharmaceutical care in vaccination: A review of literature.
#519	MEDLINE_EMBASE	Impact of pharmaceutical care in cancer: A review of literature.
#520	MEDLINE_EMBASE	A systematic review of the effect of cancer treatment on work productivity of patients and caregivers.
#521	MEDLINE_EMBASE	A systematic review on the role and impact of the clinical transplant pharmacist.
#522	MEDLINE_EMBASE	Cost and dosage of biological therapies in clinical practice of rheumatic diseases.

#523	MEDLINE_EMBASE	A Review of US Drug Costs Relevant to Medicare, Medicaid, and Commercial Insurers Post-Affordable Care Act Enactment, 2010-2016.
#525	MEDLINE_EMBASE	Economic evaluation of chronic disease self-management for people with diabetes: A systematic review.
#527	MEDLINE_EMBASE	Emergency hospital drug cabinet systems managed by hospital pharmacists and er physicians: A healthcare management model to reduce costs.
#528	MEDLINE_EMBASE	Pharmacist prescribing practices in a clinical pharmacy cardiac risk service.
#529	MEDLINE_EMBASE	Prescribing pharmacists in the ambulatory care setting: Experience at the University of North Carolina Medical Center.
#530	MEDLINE_EMBASE	Elevating pharmacists' scope of practice through a health-system clinical privileging process.
#531	MEDLINE_EMBASE	A metaanalysis of interventions to improve adherence to lipid-lowering medication.
#532	MEDLINE_EMBASE	Big data: Implications for health system pharmacy.
#533	MEDLINE_EMBASE	Community and hospital pharmaceutical care systematic review. Control cardiovascular risk in Spain and other countries. [Spanish]
#534	MEDLINE_EMBASE	Indicators in evaluating financial and economic performance of pharmacy: A systematic review.
#536	MEDLINE_EMBASE	Adherence in pediatric kidney transplant recipients: solutions for the system.
#537	MEDLINE_EMBASE	Post discharge medicines use review (dMUR) service for older patients: Cost-savings from community pharmacist interventions.
#539	MEDLINE_EMBASE	Economic Evaluation of Hospital and Community Pharmacy Services: A Review of the Literature (2010-2015).
#540	MEDLINE_EMBASE	Community pharmacy interventions for public health priorities: a systematic review of community pharmacy-delivered smoking, alcohol and weight management interventions.
#541	MEDLINE_EMBASE	A cluster randomised control trial to evaluate the effectiveness and cost-effectiveness of the Italian medicines use review (I-MUR) for asthma patients.
#542	MEDLINE_EMBASE	Effectiveness of pharmacy-based needle/syringe exchange programme for people who inject drugs: a systematic review and meta-analysis.
#543	MEDLINE_EMBASE	Pharmacy Benefits Management in the Veterans Health Administration Revisited: A Decade of Advancements, 2004-2014.
#545	MEDLINE_EMBASE	Management of multiple sclerosis and the integration of related specialty pharmacy programs within health systems.
#546	MEDLINE_EMBASE	Specialty pharmacy for hematologic malignancies.
#547	MEDLINE_EMBASE	Adherence With Therapeutic Regimens: Behavioral and Pharmacoeconomic Perspectives.
#548	MEDLINE_EMBASE	ECONOMIC ISSUES IN THERAPEUTIC DRUG MONITORING.
#550	DARE_NHESEED_HTA	Home care nursing.
#551	DARE_NHESEED_HTA	Economic evaluation of enoxaparin for the prevention of venous thromboembolism in acutely ill medical patients.
#552	DARE_NHESEED_HTA	Peginterferon alfa-2a versus peginterferon alfa-2b as initial treatment of hepatitis C virus infection: a cost-utility analysis from the perspective of the Veterans Affairs health care system.
#554	DARE_NHESEED_HTA	The economics of a pharmacy-based central intravenous additive service for paediatric patients.
#558	DARE_NHESEED_HTA	Hospital clinical pharmacy research in the UK: a review and annotated bibliography.
#559	DARE_NHESEED_HTA	The impact of pharmacist interventions on health-related quality of life.
#560	DARE_NHESEED_HTA	Systematic review of the use of patients' own medications in acute care institutions.
#561	DARE_NHESEED_HTA	Effectiveness of interventions by community pharmacists to improve patient adherence to chronic medication: a systematic review.
#562	DARE_NHESEED_HTA	A cost-effectiveness analysis of a community pharmacist-based smoking cessation programme in Thailand.
#563	DARE_NHESEED_HTA	Cost-effectiveness of pharmacy and group behavioural support smoking cessation services in Glasgow.
#564	DARE_NHESEED_HTA	An estimation of the long-term clinical and economic benefits of insulin lispro in type 1 diabetes in the UK.
#565	DARE_NHESEED_HTA	The potency of team-based care interventions for hypertension: a meta-analysis.
#566	DARE_NHESEED_HTA	US Pharmacists' Effect as Team Members on Patient Care: Systematic Review and Meta-Analyses.
#567	DARE_NHESEED_HTA	Effectiveness of pharmacist-participated warfarin therapy management: a systematic review and meta-analysis.
#568	DARE_NHESEED_HTA	Effect of pharmaceutical care programs on glycemic control in patients with diabetes mellitus: a meta-analysis of randomized controlled trials.
#570	DARE_NHESEED_HTA	One-year outcomes and a cost-effectiveness analysis for smokers accessing group-based and pharmacy-led cessation services.
#571	DARE_NHESEED_HTA	Pyxis and medDISPENSE automated medication dispensing systems: a review of the clinical benefits and harms, cost-effectiveness, and guidelines for use.
#573	DARE_NHESEED_HTA	Is Accelerated Partner Therapy (APT) a cost-effective alternative to routine patient referral partner notification in the UK? Preliminary cost-consequence analysis of an exploratory trial.
#575	DARE_NHESEED_HTA	Interventions promoting adherence to cardiovascular medicines.

#577	DARE_NHESEED_HTA	Pharmacist interventions to improve cardiovascular disease risk factors in diabetes: a systematic review and meta-analysis of randomized controlled trials.
#578	DARE_NHESEED_HTA	The impact of HIV clinical pharmacists on HIV treatment outcomes: a systematic review.
#580	DARE_NHESEED_HTA	Are pharmacy-based minor ailment schemes a substitute for other service providers? A systematic review.
#581	DARE_NHESEED_HTA	Pharmacist services provided in general practice clinics: a systematic review and meta-analysis.
#584	TUFTS	Cost-effectiveness of statins for primary prevention in patients newly diagnosed with type 2 diabetes in the Netherlands.
#585	TUFTS	Feasibility and cost-effectiveness of stroke prevention through community screening for atrial fibrillation using iPhone ECG in pharmacies. The SEARCH-AF study.
#586	TUFTS	Economic evaluation of posaconazole versus fluconazole or itraconazole in the prevention of invasive fungal infection in high-risk neutropenic patients in Sweden.
#587	TUFTS	Improving adherence to lipid-lowering therapy in a community pharmacy intervention program: a cost-effectiveness analysis.
#588	TUFTS	Economic evaluation of aclidinium bromide in the management of moderate to severe COPD: an analysis over 5 years.
#589	TUFTS	Evaluation of the cost-utility of insulin degludec vs insulin glargine in Sweden.
#590	TUFTS	Cost-Effectiveness Analysis of Direct-Acting Antiviral Therapy for Treatment-Naive Patients With Chronic Hepatitis C Genotype 1 Infection in the Veterans Health Administration.
#591	TUFTS	A UK Analysis of the Cost-Effectiveness of Humalog Mix75/25 and Mix50/50 Versus Long-Acting Basal Insulin.
#592	TUFTS	The clinical and economic burden of poor adherence and persistence with osteoporosis medications in Ireland.
#593	TUFTS	Long-term cost-utility analysis of exenatide once weekly versus insulin glargine for the treatment of type 2 diabetes patients in the US.
#594	TUFTS	Health and economic outcomes for exenatide once weekly, insulin, and pioglitazone therapies in the treatment of type 2 diabetes: a simulation analysis.
#595	TUFTS	Cost-utility and budget impact analysis of primary prevention with alendronate of osteoporotic hip fractures in Catalonia.
#596	TUFTS	A long-term analysis evaluating the cost-effectiveness of biphasic insulin lispro mix 75/25 and mix 50/50 versus long-acting basal insulin analogs in the United States.
#597	TUFTS	Incremental cost-effectiveness of the pre- and post-bevacizumab eras of metastatic colorectal cancer therapy in British Columbia, Canada.
#598	TUFTS	Evaluating the cost-effectiveness of reduced mild hypoglycaemia in subjects with Type 1 diabetes treated with Insulin detemir or NPH insulin in Denmark, Sweden, Finland and the Netherlands.
#599	TUFTS	The clinical benefit and cost-effectiveness of human papillomavirus vaccination for adult women in the Netherlands.
#600	TUFTS	Cost-utility analysis of a pharmacy-led self-management programme for patients with COPD.
#601	TUFTS	Cost-effectiveness of exenatide versus insulin glargine in Spanish patients with obesity and type 2 diabetes mellitus.
#602	TUFTS	Incremental cost-effectiveness of various monthly doses of vardenafil.
#603	TUFTS	Cost-effectiveness analysis of tapentadol immediate release for the treatment of acute pain.
#604	TUFTS	The cost effectiveness of rapid-acting insulin aspart compared with human insulin in type 2 diabetes patients: an analysis from the Japanese third-party payer perspective.
#606	TUFTS	Cost-effectiveness analysis of a rural telemedicine collaborative care intervention for depression.
#607	TUFTS	A cost-effectiveness analysis to illustrate the impact of cost definitions on results, interpretations and comparability of pharmacoeconomic studies in the US.
#608	TUFTS	Is INTERdisicplinary COMmunity-based COPD management (INTERCOM) cost-effective?
#609	TUFTS	Cost-effectiveness of aliskiren in type 2 diabetes, hypertension, and albuminuria.
#610	TUFTS	Short-Term Cost and Health Consequences of Duodenal Levodopa Infusion in Advanced Parkinson's Disease in Sweden: An Exploratory Study.
#612	TUFTS	Cost-effectiveness of switching to biphasic insulin aspart in poorly-controlled type 2 diabetes patients in China.
#613	TUFTS	Combination polypharmacy for cardiovascular disease prevention in men: a decision analysis and cost-effectiveness model.
#614	TUFTS	Cost-effectiveness of intensified versus conventional multifactorial intervention in type 2 diabetes: results and projections from the Steno-2 study.
#615	TUFTS	Economic evaluation of standing order programs for pneumococcal vaccination of hospitalized elderly patients.
#616	TUFTS	Home-based medication review in older people: is it cost effective?
#617	TUFTS	Exenatide versus insulin glargine in patients with type 2 diabetes in the UK: a model of long-term clinical and cost outcomes.
#618	TUFTS	Therapy conversion to insulin detemir among patients with type 2 diabetes treated with oral agents: a modeling study of cost-effectiveness in the United States.
#619	TUFTS	Cost-effectiveness of the two-compound formulation calcipotriol and betamethasone dipropionate compared with commonly used topical treatments in the management of moderately severe plaque psoriasis in Scotland.
#620	TUFTS	Cost-effectiveness of low-molecular-weight heparin for secondary prophylaxis of cancer-related venous thromboembolism.

#621	TUFTS	Cost-effectiveness of topiramate in migraine prevention: results from a pharmacoeconomic model of topiramate treatment.
#622	TUFTS	Cost-effectiveness of low-molecular-weight heparin for treatment of pulmonary embolism.
#623	TUFTS	Prevention with tamoxifen or other hormones versus prophylactic surgery in BRCA1/2-positive women: a decision analysis.
#624	TUFTS	Low-molecular-weight heparins compared with unfractionated heparin for treatment of acute deep venous thrombosis. A cost-effectiveness analysis.
#625	TUFTS	A pharmacoeconomic model to aid in the allocation of ambulatory clinical pharmacy services.
#626	TUFTS	Cost-utility analysis of a multidisciplinary strategy to manage osteoarthritis of the knee: economic evaluation of a cluster randomized controlled trial study.
#627	TUFTS	Cost effectiveness of a pharmacist-led information technology intervention for reducing rates of clinically important errors in medicines management in general practices (PINCER).
#628	TUFTS	Cost-utility analysis of a pharmacotherapy follow-up for elderly nursing home residents in Spain.
#629	TUFTS	Incremental cost effectiveness of pharmacist-managed erythropoiesis-stimulating agent clinics for non-dialysis-dependent chronic kidney disease patients.
#630	TUFTS	Cost-effectiveness of pharmacist-participated warfarin therapy management in Thailand.
#631	TUFTS	Cost-effectiveness of a community pharmacist intervention in patients with depression: a randomized controlled trial (PRODEFAR Study).
#632	TUFTS	A multidisciplinary support programme increases the efficiency of pegylated interferon alfa-2a and ribavirin in hepatitis C.
#633	TUFTS	Cost-Effectiveness of a Community Pharmacist-Led Sleep Apnea Screening Program - A Markov Model.
#634	TUFTS	A Markov model of the cost-effectiveness of pharmacist care for diabetes in prevention of cardiovascular diseases: evidence from Kaiser Permanente Northern California.
#635	TUFTS	Health economic evaluation of the Lund Integrated Medicines Management Model (LIMM) in elderly patients admitted to hospital.
#636	TUFTS	Cost-effectiveness of pharmacist-provided treatment of adult pharyngitis.
#637	TUFTS	A cost-effectiveness analysis of an in-hospital clinical pharmacist service.
#638	TUFTS	Model-based cost-effectiveness analysis of interventions aimed at preventing medication error at hospital admission (medicines reconciliation).
#639	TUFTS	Economic analysis of albumin-bound paclitaxel for the treatment of metastatic breast cancer.
#640	TUFTS	The cost effectiveness of anticoagulation management services for patients with atrial fibrillation and at high risk of stroke in the US.
#641	TUFTS	Cost analysis and cost-benefit analysis of a medication review with follow-up service in aged polypharmacy patients.
#642	TUFTS	Massachusetts Health Reform Cost Less and Was More Effective for Uninsured Individuals With Venous Thromboembolism: A Cost-Effectiveness Analysis.
#643	TUFTS	Cost-effectiveness of adding carfilzomib to lenalidomide and dexamethasone in relapsed multiple myeloma from a US perspective.
#644	TUFTS	Cost-Effectiveness Analysis of Incretin Therapy for Type 2 Diabetes in Spain: 1.8 mg Liraglutide Versus Sitagliptin.
#645	TUFTS	Cost-utility analysis of a medication review with follow-up service for older adults with polypharmacy in community pharmacies in Spain: the conSIGUE program.
#646	TUFTS	The four or more medicines (FOMM) support service: results from an evaluation of a new community pharmacy service aimed at over-65s.
#647	TUFTS	Cost-effectiveness of Collaborative Care for Depression in Human Immunodeficiency Virus Clinics.
#648	TUFTS	Cost-Effectiveness Analysis of Treating Acute Promyelocytic Leukemia Patients With Arsenic Trioxide and Retinoic Acid in the United States.
#649	TUFTS	Chronic obstructive pulmonary disease case finding by community pharmacists: a potential cost-effective public health intervention.
#650	TUFTS	Economic analysis of insulin initiation by pharmacists in a Canadian setting: The RxING study.
#651	TUFTS	Economic evaluation of a pharmaceutical care program for elderly diabetic and hypertensive patients in primary health care: a 36-month randomized controlled clinical trial.
#652	TUFTS	Cost-effectiveness analysis of smoking-cessation counseling training for physicians and pharmacists.
#653	TUFTS	Care in the Home for the Management of Chronic Heart Failure: Systematic Review and Cost-Effectiveness Analysis.
#654	TUFTS	Pharmacist-led management of chronic pain in primary care: costs and benefits in a pilot randomised controlled trial.
#655	CDSR	Interventions to improve adherence to inhaled steroids for asthma.
#656	CDSR	Pharmacological interventions for non-alcohol related fatty liver disease (NAFLD).
#657	CDSR	Interventions to improve inhaler technique for people with asthma.
#658	CDSR	Fixed-dose combination therapy for the prevention of atherosclerotic cardiovascular diseases.
#659	CDSR	Interventions to improve antibiotic prescribing practices for hospital inpatients.
#660	CDSR	Training interventions for improving telephone consultation skills in clinicians.
#662	CDSR	Automated telephone communication systems for preventive healthcare and management of long-term conditions.
#663	CDSR	Non-medical prescribing versus medical prescribing for acute and chronic disease management in primary and secondary care.

#664	CDSR	Tools developed and disseminated by guideline producers to promote the uptake of their guidelines.
#665	CDSR	Public stewardship of private for-profit healthcare providers in low- and middle-income countries.
#666	CDSR	Home telemonitoring and remote feedback between clinic visits for asthma.
#667	CDSR	Remote versus face-to-face check-ups for asthma.
#668	CDSR	Organising healthcare services for persons with an intellectual disability.
#669	CDSR	Medication review in hospitalised patients to reduce morbidity and mortality.
#670	CDSR	Interventions to optimise prescribing for older people in care homes.
#671	CDSR	Interventions for dysphagia in long-term, progressive muscle disease.
#672	CDSR	Interventions for preventing unintended pregnancies among adolescents.
#673	CDSR	Systematic versus opportunistic risk assessment for the primary prevention of cardiovascular disease.
#674	CDSR	Discharge planning from hospital.
#675	CDSR	Interactive telemedicine.
#676	CDSR	Patient controlled opioid analgesia versus non-patient controlled opioid analgesia for postoperative pain.
#677	CDSR	Chronic disease management programmes for adults with asthma.
#678	CDSR	Negative pressure wound therapy for treating pressure ulcers.
#679	CDSR	Interventions for hirsutism (excluding laser and photoepilation therapy alone).
#680	CDSR	Strategies to increase the ownership and use of insecticide-treated bednets to prevent malaria.
#681	CDSR	Interventions for reducing medication errors in children in hospital.
#682	CDSR	Interventions to reduce waiting times for elective procedures.
#683	CDSR	Low dose versus high dose stavudine for treating people with HIV infection.
#684	CDSR	Interventions for enhancing medication adherence.
#685	CDSR	Antibiotic prophylaxis versus no prophylaxis for preventing infection after cesarean section.
#686	CDSR	Interventions for managing asthma in pregnancy.
#687	CDSR	Interventions to improve the appropriate use of polypharmacy for older people.
#688	CDSR	Interventions for improving the adoption of shared decision making by healthcare professionals.
#689	CDSR	Systemic safety of bevacizumab versus ranibizumab for neovascular age-related macular degeneration.
#690	CDSR	Dental auxiliaries for dental care traditionally provided by dentists.
#691	CDSR	Exit interviews to reduce turnover amongst healthcare professionals.
#692	CDSR	Intravenous magnesium sulfate for treating adults with acute asthma in the emergency department.
#693	CDSR	Interventions to improve safe and effective medicines use by consumers.
#694	CDSR	Antidepressants for smoking cessation.
#695	CDSR	Computerized advice on drug dosage to improve prescribing practice.
#696	CDSR	The effect of different methods of remuneration on the behaviour of primary care dentists.
#697	CDSR	Collaborative care approaches for people with severe mental illness.
#698	CDSR	Integrated disease management interventions for patients with chronic obstructive pulmonary disease.
#699	CDSR	Telephone counselling for smoking cessation.
#700	CDSR	Interventions for implementation of thromboprophylaxis in hospitalized medical and surgical patients at risk for venous thromboembolism.
#701	CDSR	Effectiveness and cost-effectiveness of home palliative care services for adults with advanced illness and their caregivers.
#702	CDSR	Interventions to improve professional adherence to guidelines for prevention of device-related infections.
#704	CDSR	Interventions for preventing falls in older people in care facilities and hospitals.
#705	CDSR	Primary care professionals providing non-urgent care in hospital emergency departments.
#706	CDSR	Information provision for stroke patients and their caregivers.
#707	CDSR	Printed educational materials.
#708	CDSR	Collaborative care for depression and anxiety problems.
#709	CDSR	Clinical service organisation for heart failure.
#710	CDSR	Interventions for preventing falls in older people living in the community.
#711	CDSR	Interventions for smoking cessation in hospitalised patients.
#712	CDSR	Training health professionals in smoking cessation.
#713	CDSR	Interventions for treating oral mucositis for patients with cancer receiving treatment.
#714	CDSR	Effect of outpatient pharmacists' non-dispensing roles on patient outcomes and prescribing patterns.
#715	CDSR	Interventions used to improve control of blood pressure in patients with hypertension.

#716	CDSR	Service organisation for the secondary prevention of ischaemic heart disease in primary care.
#717	CDSR	Antibiotic prophylaxis to reduce respiratory tract infections and mortality in adults receiving intensive care.
#718	CDSR	Individual patient education for people with type 2 diabetes mellitus.
#719	CDSR	Interventions for improving older patients' involvement in primary care episodes.
#720	CDSR	Telephone follow-up, initiated by a hospital-based health professional, for postdischarge problems in patients discharged from hospital to home.
#721	CDSR	Community pharmacy personnel interventions for smoking cessation.
#726	WoS	Clinical medication review in Australia: A systematic review.
#729	WoS	Community pharmacies as sites of adult vaccination: A systematic review.
#731	WoS	The impact of patient support programs on adherence, clinical, humanistic, and economic patient outcomes: a targeted systematic review.
#732	WoS	A Systematic Review of Interventions Addressing Adherence to Anti-Diabetic Medications in Patients with Type 2 Diabetes-Components of Interventions.
#733	WoS	Emergence of BCR-ABL Kinase Domain Mutations Associated with Newly Diagnosed Chronic Myeloid Leukemia: A Meta-Analysis of Clinical Trials of Tyrosine Kinase Inhibitors.
#735	WoS	Meta-analysis of the effectiveness of smoking cessation interventions in community pharmacy.
#738	WoS	Effectiveness of tobacco use cessation interventions delivered by pharmacy personnel: A systematic review.
#739	WoS	Interventions to improve osteoporosis medication adherence and persistence: a systematic review and literature appraisal by the ISPOR.
#741	WoS	Information technology interventions to improve medication safety in primary care: a systematic review.
#742	WoS	Approaches for improving continuity of care in medication management: a systematic review.
#746	WoS	Cooperation between general practitioners and pharmacists: a systematic review.
#747	WoS	Chlamydia screening interventions from community pharmacies: a systematic review.
#748	WoS	Chlamydia screening in community pharmacies: a systematic literature review of the characteristics of service users and a meta-analysis of chlamydia prevalence.
#750	WoS	Pharmacists' interventions in the management of patients with chronic kidney disease: a systematic review.
#752	WoS	Pharmacist interventions to enhance blood pressure control and adherence to antihypertensive therapy: Review and meta-analysis.
#756	WoS	The methodological quality of economic evaluations of guideline implementation into clinical practice: A systematic review of empiric studies.
#757	WoS	Pharmacists versus nonpharmacists in adverse drug event detection: A meta-analysis and systematic review.
#758	WoS	Collaborative care for depression in primary care - Making sense of a complex intervention: systematic review and meta-regression.
#759	WoS	Clinical pharmacists and inpatient medical care - A systematic review.
