# Supplementary material for: An overview of systematic reviews of economic evaluations of pharmacy-based public health interventions: addressing methodological challenges
Source: Syst Rev. 2019 Nov 11;8:272. doi: 10.1186/s13643-019-1177-3 (PMC6844055; doi:10.1186/s13643-019-1177-3)
Supplement: Supplementary file 5 — Additional file 5. Quality of included reviews in all 16 items (AMSTAR 2) – this file contains the complete quality assessment of included reviews in all 16 items of AMSTAR 2. [file 13643_2019_1177_MOESM5_ESM.pdf]

An Overview of Systematic Reviews of Economic Evaluations of Pharmacy-Based Public Health Interventions: Addressing Methodological Challenges

Additional File 5: Quality of included reviews in all 16 items (AMSTAR 2)

No.	AMSTAR 2 Item	Yes	Partial Yes	No meta-analysis	n (%)	Critical domains
1	Research question and inclusion criteria include components of PICO	All	-	-	14 (100%)	-
2	Explicit statement – review methods established prior to review and justification for deviations from protocol	[50, 55]	-	-	2 (14%)	●
3	Explained selection of study designs for inclusion	[44, 45, 46, 48, 49, 24, 50 51, 52, 53, 54, 55]	-	-	12 (86%)	-
4	Used comprehensive literature search strategy	[50]	[44, 45, 46, 47, 48, 49, 24, 51, 52, 53, 54, 55, 56]	-	14 (100%)	●
5	Performed study selection in duplicate	[44, 45, 46, 47, 48, 49, 50, 51, 53, 54, 55, 56]	-	-	12 (86%)	-
6	Performed data extraction in duplicate	[44, 45, 46, 47, 48, 50, 53 55]	-	-	8 (57%)	-
7	Provided list of excluded studies and justification for exclusions	-	-	-	0 (0%)	●
8	Described included studies in adequate detail	[49, 24 50, 51, 52, 53]	[44, 45, 46, 47, 48, 54, 55]	-	13 (93%)	-
9	Used satisfactory technique for assessing risk of bias in individual studies included in review	[50, 54, 55]	[24, 56]	-	5 (36%)	(1)
10	Reported on the sources of funding for the studies included in the review	[48, 50]	-	-	2 (14%)	-
11	Used appropriate methods for statistical combination of results if meta-analysis performed	[50, 54]	-	[44, 45, 46, 47, 48, 49, 24, 51, 52, 53, 55, 56]	2 (100%)	(2)
12	Assessed potential impact of risk of bias in individual studies on the results of meta-analysis or other synthesis	[50]	-	[44, 45, 46, 47 48, 49, 24, 51, 52, 53, 55, 56]	1 (50%)	-
13	Accounted for risk of bias in individual studies when interpreting/discussing results of review	[45, 46, 48 49, 24, 50, 53, 54, 55, 56]	-	-	10 (71%)	●
14	Provided satisfactory explanation/discussion of any heterogeneity observed in results of review	[44, 45, 46, 48, 49, 24, 50, 51, 53, 55]	-	-	10 (71%)	-
15	Carried out adequate investigation of publication bias and discussed impact on the results of review	[50]	-	[44, 45, 46, 47, 48, 49, 24, 51, 52, 53, 55, 56]	1 (50%)	(3)
16	Reported potential sources of conflict of interest, including funding received for conducting the review	[44, 45, 46, 47,48, 49, 24, 50, 51, 53, 54, 55, 56]	-	-	13 (93%)	-

(1) Not considered: instruments for reviews that include nonrandomized trials were published in 2016 (ROBINS-I) and 2017 (Suggested Risk of Bias criteria for EPOC Reviews) only

(2) Not considered: most reviews are narrative

(3) Not considered: included reviews have in average 8 studies (<10)
